# Transfer learning for subject-independent motor imagery EEG classification using convolutional relational networks

**DOI:** 10.3389/fnins.2025.1691929

**Published:** 2026-01-02

**Authors:** Zhenis Otarbay, Abzal Kyzyrkanov

**Affiliations:** 1 Department of Robotics Engineering, Nazarbayev University, Astana, Kazakhstan; 2 School of Software Engineering, Astana IT University, Astana, Kazakhstan

**Keywords:** electroencephalography (EEG), motor imagery, brain-computer interface (BCI), transfer learning, convolutional relational networks, subject-independent classification, neural signal processing

## Abstract

Motor imagery (MI) based electroencephalography (EEG) classification is central to brain-computer interface (BCI) research but practical deployment remains challenging due to poor generalization across subjects. Inter-individual variability in neural activity patterns significantly limits the development of subject-independent BCIs for healthcare and assistive technologies. To address this limitation, we present a transfer learning framework based on Convolutional Relational Networks (ConvoReleNet) designed to extract subject-invariant neural representations while minimizing the risk of catastrophic forgetting. The method integrates convolutional feature extraction, relational modeling, and lightweight recurrent processing, combined with pretraining on a diverse subject pool followed by conservative fine-tuning. Validation was conducted on two widely used benchmarks, BNCI IV-2a (four-class motor imagery) and BNCI IV-2b (binary motor imagery), to evaluate subject-independent classification performance. Results demonstrate clear improvements over training from scratch: accuracy on BNCI IV-2a increased from 72.22 (±20.49) to 79.44% (±11.09), while BNCI IV-2b improved from 75.10 (±17.17) to 83.85% (±10.30). The best-case performance reached 87.55% on BNCI IV-2a with Tanh activation and 83.85% on BNCI IV-2b with ELU activation, accompanied by reductions in inter-subject variance of 45.9 and 40.0%, respectively. These findings establish transfer learning as an effective strategy for subject-independent MI-EEG classification. By enhancing accuracy, reducing variability, and maintaining computational efficiency, the proposed framework strengthens the feasibility of robust and user-friendly BCIs for rehabilitation, clinical use, and assistive applications.

## 1 Introduction

Advances in electroencephalography (EEG)-based brain-computer interfaces (BCIs) increasingly depend on artificial intelligence (AI) methods capable of extracting informative patterns from complex, noisy neural signals. The same AI techniques that have enhanced intelligent robotics, perception, and decision-making are now being adapted to improve neurotechnology. For instance, AI-driven models have achieved robust perception and control in autonomous mobile systems (Yedilkhan et al., 2024), cooperative multi-robot coordination (Kyzyrkanov et al., 2023, 2024), and automatic number-plate recognition using edge-computing vision frameworks (Seitbattalov et al., 2022, 2023). Deep learning has further advanced real-world safety monitoring through personal protective equipment detection (Barlybayev et al., 2024), improved fairness in educational assessment via fuzzy logic (Barlybayev et al., 2025), and enhanced environmental modeling using wavelet-neural networks (Malekpourheydari et al., 2022). These achievements collectively illustrate how AI's adaptability and precision can be leveraged to decode neural activity for reliable and generalizable MI-BCI applications.

Within the field of neurotechnology, AI has played an especially transformative role. Machine learning and neural network models have been adopted to enhance brain-computer interfaces (BCIs), including systems based on event-related potentials such as P300 (Otarbay et al., 2023, 2025) and visual evoked potentials (VEPs) (Keutayeva et al., 2025). Such advancements underscore the potential of AI not only in conventional automation tasks but also in enabling direct communication pathways between the human brain and external devices. Against this backdrop, Motor Imagery (MI)-based BCIs stand out as a promising avenue, leveraging EEG signals generated by the imagination of movement to provide a non-invasive means of control.

MI-BCIs are particularly significant in assistive technology for individuals with neurological disorders or severe physical impairments. By allowing users to operate external devices such as cursors, prostheses, or robotic manipulators through imagined movements, MI-BCIs provide a natural and intuitive communication channel. Their importance extends beyond clinical rehabilitation to broader applications in human-computer interaction, automation, and cognitive engagement. In medical contexts, they empower patients affected by stroke, spinal cord injury, or degenerative diseases such as amyotrophic lateral sclerosis (ALS) to regain autonomy. Their non-invasive reliance on electroencephalography (EEG) avoids surgical risks while maintaining accessibility, and their use in neurorehabilitation has shown promise in promoting neural plasticity and recovery (Li and Xu, 2024). Thus, MI-BCIs occupy a pivotal position at the convergence of neuroscience, artificial intelligence, and human-centered technology, underscoring the urgency of addressing their current limitations.

From a neurophysiological standpoint, motor imagery elicits activity patterns in cortical regions—particularly the sensorimotor cortex—producing characteristic event-related desynchronization (ERD) and synchronization (ERS) responses in the μ (8–12 Hz) and β (13–30 Hz) frequency bands. These rhythmic modulations represent the core discriminative features exploited in MI-EEG decoding and provide a biological foundation for feature extraction and transfer-learning strategies.

Despite their promise, the development of robust and practical MI-BCIs is constrained by several challenges. A typical EEG-based BCI pipeline involves signal acquisition, preprocessing, feature extraction, and classification to map brain signals to control commands (Jaipriya and Sriharipriya, 2025). Accurate decoding is hindered by the low signal-to-noise ratio of EEG data and by its variability across subjects and sessions (Otarbay and Kyzyrkanov, 2025). This variability forces many systems to rely on subject-specific calibration, which requires extensive labeled data and imposes a significant barrier to scalability and user-friendliness (Jaipriya and Sriharipriya, 2025). To mitigate this, the research community has increasingly pursued subject-independent approaches.

Convolutional Neural Networks (CNNs) have emerged as a promising solution by automatically learning spatio-temporal EEG features without relying on manual feature extraction (Jaipriya and Sriharipriya, 2025). They often outperform traditional approaches such as Support Vector Machines (SVMs) and Common Spatial Patterns (CSP). Transfer learning (TL) has further strengthened MI-BCI development by reusing knowledge across subjects, thereby reducing the demand for large calibration datasets (Otarbay and Kyzyrkanov, 2025). Fine-tuning of pre-trained CNNs is one of the most widely adopted TL strategies (Li X. et al., 2024), yet its optimal implementation for subject-independent MI-EEG classification remains unclear. Key questions–such as which network layers to retrain, how much data to collect, and how to balance learning rates–are unresolved, and inappropriate fine-tuning risks either catastrophic forgetting or insufficient adaptation (Wu et al., 2020).

Recent research has broadened this scope by introducing domain-alignment, adaptive re-weighting, and meta-transfer frameworks for MI-EEG decoding, achieving improved cross-subject stability and session-invariant performance (Zhang et al., 2023; Zhao et al., 2024, 2025a). These studies demonstrate that transfer-learning strategies remain an evolving field, motivating further exploration of controlled fine-tuning policies for reliable generalization.

This persistent challenge, often termed the “last mile” problem, continues to limit the deployment of practical subject-independent MI-BCIs (Zhang et al., 2023). Addressing it requires systematic evaluation of fine-tuning strategies rather than *ad-hoc* experimentation. In this work, we investigate fine-tuning schedules for CNN-based MI-BCIs using rigorous Leave-One-Subject-Out (LOSO) cross-validation. Experiments on the BCI Competition IV-2a dataset show that a conservative fine-tuning strategy improves accuracy from 63.79 to 86.21% and reduces inter-subject variance from 14.05 to 6.94%, demonstrating both higher performance and greater reliability. Further results on the IV-2b dataset reveal that limiting fine-tuning with lower learning rates and fewer epochs mitigates catastrophic forgetting, increasing accuracy from 74.36 to 77.64%. Finally, an architectural comparison highlights the role of activation functions, where ReLU improves classification accuracy by more than two percentage points compared to ELU. Together, these results provide empirically grounded guidelines for fine-tuning in subject-independent EEG decoding. In this study, the term “relational” in Convolutional Relational Network denotes the model's capacity to capture inter-channel and temporal dependencies among EEG features through multi-branch convolutional fusion and the self-attention mechanism of the Transformer encoder, rather than implying a separate relational or graph-based module.

By systematically analyzing fine-tuning strategies across datasets and architectures, this study contributes toward more reliable, calibration-efficient, and practical MI-BCIs. These findings advance the field of subject-independent EEG decoding and support the transition of BCIs from laboratory prototypes to real-world applications.

Although transfer learning has been previously explored for motor-imagery EEG decoding, most have focused on single-architecture fine-tuning or dataset-specific adaptation. In contrast, the present work introduces a unified hybrid framework that combines multi-branch convolutional feature extraction, Transformer-based relational encoding, and sequential modeling through BiLSTM layers. This design enables the joint learning of spatial, temporal, and cross-subject dependencies, facilitating more reliable knowledge transfer across heterogeneous EEG datasets. Furthermore, the study systematically analyzes fine-tuning depth, learning-rate adaptation, and activation selection, offering empirically grounded insights into subject-independent transfer learning for MI-BCIs.

While hybrid CNN-RNN or CNN-Transformer combinations have been proposed previously, their integration has often been partial or dataset-specific. The present framework unifies convolutional, attention-based, and recurrent components within a single architecture to jointly capture spatial, temporal, and relational dependencies, thereby balancing expressive power with interpretability.

## 2 Literature review

### 2.1 Deep learning architectures for motor imagery EEG classification

Deep learning, and in particular Convolutional Neural Networks (CNNs), has transformed the field of motor imagery (MI) EEG classification. CNNs offer the advantage of end-to-end learning, where feature extraction and classification are jointly optimized, thereby reducing reliance on manual feature engineering that is often subjective and labor-intensive. By exploiting convolutional filters, CNNs effectively capture both spatial correlations across EEG channels and temporal dynamics in neural activity. This architectural property makes them computationally efficient and well-suited for high-dimensional EEG signals, which are typically noisy and complex in structure.

The application of CNNs in MI-EEG has evolved through several important phases. Early studies demonstrated their superiority over conventional machine learning methods, achieving substantially improved accuracies even for subjects considered “BCI inefficient.” For example, deep CNN-based approaches applied to BCI Competition IV Dataset 2a achieved average accuracies as high as 97.61% (Tengis et al., 2023). To further improve robustness, multi-branch and multi-scale CNNs were proposed, which process EEG signals at multiple temporal and spatial resolutions. The multi-scale convolutional network MS-AFM (MACNN) integrated inception and residual structures and achieved an average recognition rate of 86.03% on the BCI IV 2a dataset (Atla and Sharma, 2025). Similarly, EEGNet Fusion V2, a multi-branch 2D CNN, attained accuracies of 89.6 and 87.8% for real and imagined motor activity, and 74.3 and 84.1% on BCI IV-2a and IV-2b datasets, respectively (Chowdhury et al., 2023).

Recent works have extended these architectures by coupling CNNs with Transformers and recurrent structures. CLT-Net, for instance, integrates CNNs with LSTM and Transformer modules, producing accuracies of 83.02% on BCI IV 2a and 87.11% on BCI IV 2b (Gu et al., 2025). Similarly, attention-based CNN models such as CIACNet, which combines convolutional attention with stochastic pooling, achieved 85.15% accuracy on BCI IV 2a and 90.05% on BCI IV 2b (Liao et al., 2025b). More recently, transformer-driven hybrids like MSCFormer (Zhao et al., 2025b) and EEGEncoder (Liao et al., 2025a) have shown competitive results by fusing multi-scale CNN filters with self-attention, achieving up to 88% accuracy on BCI IV-2b. Hybrid CNN-GRU models (Bouchane et al., 2025) and hybrid deep-belief frameworks (Mathiyazhagan and Devasena, 2025) also illustrate that temporal modeling remains an active area, with some methods reporting four-class accuracies exceeding 95%.

More recent studies have continued this trend toward attention-enhanced and transformer-integrated CNN frameworks for MI-EEG decoding. CTNet, a convolutional-Transformer network, demonstrated that combining convolutional feature extraction with self-attention markedly improves spatial-temporal representation learning and classification robustness across subjects (Zhao et al., 2024). Similarly, TCANet introduced a temporal convolutional attention mechanism that strengthens temporal dependency modeling and further refines feature alignment for MI-EEG tasks (Zhao et al., 2025a). These architectures exemplify the ongoing evolution toward hybrid attention-driven models that balance interpretability, accuracy, and computational efficiency.

Alongside these developments, compact CNN architectures have been proposed to balance accuracy and efficiency. EEGNet remains a benchmark due to its small size, with reported accuracies of 77.12 and 86.55% on BCI IV 2a and 2b, respectively (Lawhern et al., 2018; Wu et al., 2025). End-to-end raw EEG approaches, such as NF-EEG (Arı and Taçgın, 2024), further eliminate preprocessing, pushing generalization without handcrafted features. At the same time, more complex architectures such as EEGConformer have demonstrated slightly higher subject-independent accuracies (72.41%), but at a significant computational cost. This tension between lightweight yet robust models versus highly complex networks highlights a persistent research gap: many state-of-the-art methods achieve excellent performance under constrained conditions but remain impractical for resource-limited or real-time BCI deployments.

### 2.2 Transfer learning paradigms in MI-EEG classification

The scarcity of subject-specific data and high inter-subject variability remain key obstacles in EEG-based BCIs. Transfer learning (TL) offers a means to mitigate these issues by leveraging knowledge from a source domain, such as data from other subjects, to improve model performance in a target domain with limited data (Wu et al., 2020; Zhang et al., 2020). In MI-EEG applications, TL enables the adaptation of pre-trained models across subjects, thereby reducing the cost and effort of calibration while improving generalization (Sun et al., 2022; Xu et al., 2021; Kwon et al., 2020; Autthasan et al., 2021).

Recent advances emphasize cross-subject and multi-source TL. Semi-supervised multi-source transfer (SSMT) (Zhang et al., 2024) leverages multiple labeled sources with unlabeled target data to align distributions and showed consistent gains on BCI-IV 2a. A three-stage TL strategy (TSTL) employing optimal transport, feature adaptation, and adaptive fine-tuning was introduced in Li J. et al. (2024), improving robustness across subject sessions. Beyond subject-to-subject adaptation, task-to-task TL (Gwon and Ahn, 2024) demonstrated that information from motor execution can be successfully transferred to motor imagery, yielding 86% accuracy. In addition, adaptive deep feature representations (ADFR) (Liang et al., 2024) integrate MMD-based alignment with entropy minimization, producing stable improvements in unsupervised cross-subject decoding.

These studies collectively highlight TL as an indispensable tool for MI-EEG, but they also underscore limitations. Performance gains are often achieved through complex pipelines, which may be computationally demanding and dataset-specific. Furthermore, many methods assume availability of rich source data, which is not always feasible in practical BCI deployment. This creates an opportunity for streamlined frameworks, such as ours, which achieve consistent improvements while maintaining efficiency.

### 2.3 Fine-tuning strategies for subject-independent MI-EEG

Fine-tuning constitutes one of the most effective TL strategies for adapting CNN-based models to new users. By initializing from pre-trained parameters and adapting them to target-specific EEG data, fine-tuning improves convergence and generalization relative to training from scratch (Zhang et al., 2021; Chang et al., 2022; Wang et al., 2022). Studies have demonstrated that even modest amounts of subject-specific data substantially improve recognition performance when fine-tuning is employed (Kwon et al., 2020; Zhang et al., 2021; Dong et al., 2023).

Enhancements to fine-tuning include Euclidean Alignment (EA), which reduces distributional divergence between domains and accelerates training convergence (Kwon et al., 2020). Meta-learning frameworks provide another direction: subject-independent meta-learning (Ng and Guan, 2024) prepares models to adapt rapidly to unseen subjects, even with zero calibration data. Similarly, continual fine-tuning across longitudinal sessions (Jiang et al., 2024; Liu et al., 2025) ensures robustness to session variability, while online test-time adaptation (OTTA) dynamically updates models during deployment (Han et al., 2023; Keutayeva et al., 2024). Evolutionary optimization methods have also been explored; for instance, genetic algorithm-based fine-tuning (Vishnupriya et al., 2024) optimized hyperparameters and layer-freezing strategies, leading to notable subject-specific accuracy gains.

Hybrid methods combining domain adaptation with fine-tuning reach the current state-of-the-art, surpassing 93% in some cross-subject benchmarks (Li and Xu, 2024; Ren et al., 2024; Ma et al., 2024). However, the variability of results across datasets reveals an unresolved challenge: aggressive fine-tuning may lead to catastrophic forgetting, while conservative approaches risk underfitting. The optimal strategy therefore depends on balancing adaptation to target data while retaining generalized features–a balance directly addressed in our proposed framework.

### 2.4 Current challenges in CNN-based transfer learning for MI-EEG

Despite progress, CNN-based TL approaches still face persistent challenges. Deep and hybrid models require large training datasets and considerable computational resources, raising concerns about scalability (Keutayeva et al., 2024; Jaipriya and Sriharipriya, 2025). Data scarcity remains a fundamental bottleneck: even the largest MI-EEG datasets include only a few dozen subjects, with variability in electrode montages and protocols making cross-dataset training difficult (Zhang et al., 2020; Wang et al., 2024).

A further limitation is BCI inefficiency, where 15%–30% of users cannot generate sufficiently discriminable motor imagery patterns (Kwon et al., 2020; Autthasan et al., 2021; Chang et al., 2022). While TL mitigates inter-subject variability, it does not fully resolve this issue, particularly in BCI-naïve populations. Moreover, real-world deployment introduces environmental noise and motion artifacts that reduce robustness outside laboratory conditions (Hu et al., 2024; Keutayeva et al., 2024). Wearable BCI systems with fewer electrodes highlight this trade-off: recent work (Rao et al., 2024) demonstrated that four-channel devices can approach the performance of full-cap systems, but accuracy remains lower for complex MI tasks.

Another barrier is the limited repertoire of separable MI tasks, which restricts the number of reliable commands. More complex paradigms such as finger-level MI reduce accuracy significantly (Penava and Buettner, 2023). Data augmentation via generative models offers one potential solution, but quality control is critical. GAN- or simulation-based augmentation (Galvan et al., 2024) can improve training, but low-quality synthetic signals risk degrading performance. These challenges illustrate a clear gap: the field requires architectures that not only achieve high accuracy but also maintain robustness with limited data, reduced channels, and under realistic deployment conditions. Our proposed framework addresses these challenges by combining efficient architectural design with conservative fine-tuning strategies that preserve generalizable features across users and sessions.

### 2.5 Synthesis and research gap

The trajectory of MI-EEG research demonstrates clear progress in leveraging CNNs, hybrid architectures, and transfer learning strategies to enhance subject-independent classification. Yet, several challenges persist. Deep and attention-driven models often achieve high performance at the expense of computational efficiency, limiting their applicability in resource-constrained or real-time BCI systems. Transfer learning methods, while powerful, remain vulnerable to catastrophic forgetting and often require elaborate pipelines that are not easily deployable. Fine-tuning strategies show promise, but their effectiveness varies across datasets, revealing the difficulty of balancing generalization with subject-specific adaptation. Moreover, persistent issues such as BCI inefficiency, inter-session variability, and robustness under noisy or wearable conditions highlight that current solutions remain far from optimal for practical applications.

These gaps underscore the need for frameworks that integrate the strengths of transfer learning with architectural efficiency and controlled fine-tuning. By addressing both accuracy and stability across diverse users while maintaining computational feasibility, such an approach directly responds to the limitations identified in recent literature. The proposed ConvoReleNet framework, coupled with a conservative fine-tuning strategy, is designed to fill this space by providing robust subject-independent MI-EEG decoding without incurring the trade-offs observed in existing methods.

## 3 Materials and methods

The methodological framework of this study was designed to rigorously evaluate the effectiveness of transfer learning for subject-independent motor imagery EEG classification. To ensure transparency and reproducibility, all methodological choices are explicitly described, covering the datasets employed, the preprocessing pipeline, the architectural design of the proposed deep neural network, the structure of the experimental protocol, and the metrics used for performance assessment. Two benchmark datasets from the BCI Competition IV were selected as they represent widely recognized testbeds for motor imagery classification and allow for direct comparison with existing approaches. The analysis begins with systematic preprocessing and subject-level data partitioning, followed by the implementation of a hybrid deep learning architecture, ConvoReleNet, which integrates convolutional, transformer, and recurrent components to capture spatial, temporal, and contextual dependencies in EEG signals.

The experimental design was structured to disentangle the contributions of transfer learning, fine-tuning strategies, and activation functions, while also validating the approach on an independent dataset. To contextualize performance gains, a traditional machine learning baseline was included, thereby establishing a reference point against which improvements from the proposed framework can be measured. Throughout the methodology, the central objective was to evaluate not only the raw classification accuracy but also the stability of performance across subjects, since inter-subject variability remains a critical bottleneck in the development of generalizable BCI systems. Figures and schematic diagrams are provided to illustrate the workflow, the data partitioning procedure, and the architecture, ensuring that the methodological process can be clearly understood and independently reproduced.

### 3.1 Dataset description

The experimental evaluation in this study is based on two publicly available datasets from the BCI Competition IV, namely BNCI 2008-IV-2a and BNCI 2008-IV-2b (Tangermann et al., 2012). These corpora have become the de facto benchmark for motor imagery EEG classification, as they provide standardized protocols and controlled acquisition conditions while simultaneously exposing the challenges of high inter-subject variability. The IV-2a dataset contains electroencephalographic recordings from nine subjects who performed four-class motor imagery tasks involving the left hand, right hand, both feet, and tongue. Signals were acquired from 22 scalp electrodes together with three electrooculographic (EOG) channels at a sampling rate of 250 Hz, with all trials time-locked to visual cues presented on a screen (Ang et al., 2012). In contrast, the IV-2b dataset consists of recordings from nine subjects who performed binary left-right hand motor imagery. EEG was recorded from three bipolar channels (C3, Cz, and C4) along with additional EOG channels, also sampled at 250 Hz.

The two datasets complement each other in terms of channel configuration and task complexity. IV-2b, with its reduced sensor setup and binary design, is particularly well suited for controlled exploration of transfer learning strategies and architectural variants, and thus was employed as the development set. IV-2a, with its higher dimensionality and four-class design, provides a more demanding benchmark for assessing generalization and therefore served as the validation set. By structuring the methodology in this manner, the analysis disentangles dataset-specific optimization from the evaluation of true subject-independent generalization, which is a central objective in motor imagery BCI research.

Electrooculographic (EOG) channels were not used as model inputs. Instead, they were utilized during preprocessing for artifact detection and manual inspection. Trials showing large-amplitude EOG deflections (exceeding ±100 μV) were excluded prior to filtering, ensuring that residual ocular artifacts did not contaminate EEG-based feature extraction.

### 3.2 Preprocessing and data splitting

To ensure consistency across both datasets and to isolate the effect of the proposed learning framework, a uniform preprocessing pipeline was applied. Each trial was first band-pass filtered between 8 and 30 Hz using a fourth-order zero-phase Butterworth filter. This frequency range is widely recognized in the motor imagery literature as it encompasses the mu and beta rhythms, which are most informative for discriminating motor-related cortical activity (Tangermann et al., 2012; Ang et al., 2012). The use of a bidirectional filtering strategy avoided phase distortions that could otherwise bias temporal dynamics relevant for classification. Following filtering, the data were standardized on a per-subject basis using the StandardScaler implementation from scikit-learn, where the transformation parameters were fit only on the training portion of the data. This strict separation prevented information leakage into validation or test sets and preserved the integrity of the evaluation.

Data partitioning was conducted independently for each subject to reflect subject-independent generalization while avoiding cross-contamination. For BNCI 2008-IV-2b, 15% of the trials were withheld as the test set, and from the remaining data, 18% was allocated to validation, with the rest used for training. For BNCI 2008-IV-2a, 20% of the trials were reserved for testing, and 25% of the residual data for validation. The proportions were selected to balance two competing requirements: reserving a sufficiently large test set to provide stable performance estimates, and maintaining adequate training data to allow effective fine-tuning of the network. Stratified splitting was employed in all cases to preserve class balance across subsets, which is critical in MI-EEG experiments where imbalanced class distributions could bias training and evaluation.

**Figure 1:**
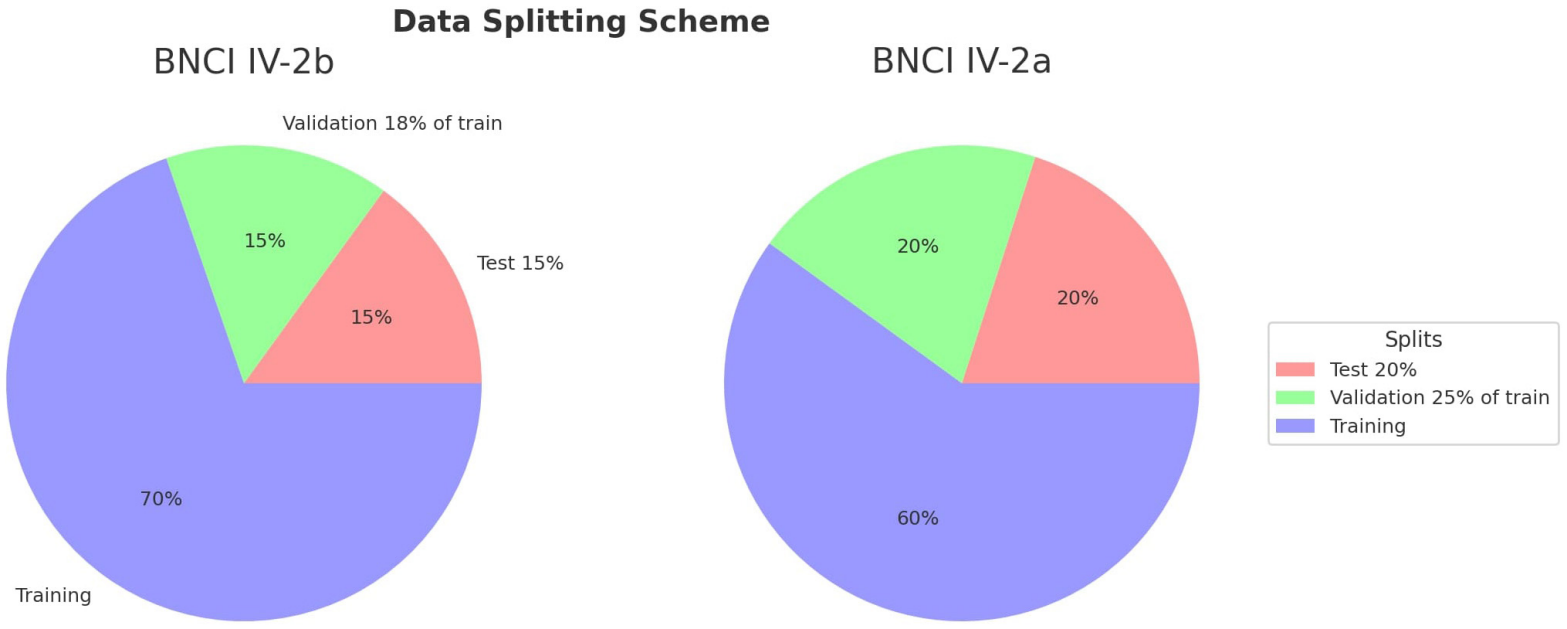
Data splitting scheme for BNCI 2008-IV-2a and BNCI 2008-IV-2b. For IV-2b, 15% of the data per subject was reserved for testing and 18% of the remainder for validation, while for IV-2a, 20% was reserved for testing and 25% of the remainder for validation. Stratification ensured balanced class distributions across all subsets.

The overall data partitioning strategy is summarized in Figure 1, which illustrates the relative proportions of training, validation, and test sets in both datasets. This scheme highlights the methodological consistency of the preprocessing and splitting pipeline, ensuring that differences in performance can be attributed to the modeling strategies rather than confounds in data handling.

Although the training-validation-test proportions were defined within each subject to maintain consistency during data preparation, all evaluations of generalization employed a subject-wise cross-validation protocol equivalent to Leave-One-Subject-Out (LOSO). In this scheme, each subject was completely excluded from training and validation when serving as the test fold, ensuring that no subject overlap occurred between partitions.

### 3.3 Model architectures

The proposed framework is based on ConvoReleNet, a hybrid deep neural network designed to capture the spatial, temporal, and contextual structure of motor imagery EEG. The model integrates convolutional, transformer, and recurrent modules in a unified architecture, allowing complementary feature extraction across different representational domains. Convolutional layers provide localized spatial-spectral representations, the transformer encoder introduces self-attention for global temporal relationships, and the recurrent component models sequential dependencies, together forming a robust representation suitable for subject-independent classification.

At the input stage, EEG data of shape (Batch, 1, *N*_*times*_, *N*_*channels*_) is processed by two parallel convolutional branches. The deep branch employs sequential temporal convolutions across two blocks, each consisting of a two-dimensional convolution, batch normalization, an activation function, max pooling, and dropout with a rate of 0.4. This path emphasizes high-level temporal-spectral representations. In contrast, the shallow branch applies a spatial convolution followed by a temporal convolution, average pooling, and dropout at the same rate of 0.4, focusing on spatial patterns over motor cortices that are especially informative in motor imagery tasks (Tangermann et al., 2012; Ang et al., 2012). Features from both branches (64 filters from the deep branch and 80 from the shallow branch) are concatenated and fused through a one-dimensional convolution, projecting them into a 128-dimensional representation.

This fused representation is then processed by a positional encoding module and passed to an eight-layer transformer encoder, configured with a model dimension of 128, four self-attention heads, and GELU activation. The encoder introduces contextual information across time and enhances discriminative feature extraction by weighting temporally relevant patterns. The transformer output is subsequently modeled by a three-layer bidirectional LSTM, with 128 hidden units per direction and a dropout rate of 0.3, which refines sequential dependencies and improves the network's ability to handle long temporal contexts. The BiLSTM outputs are mean-pooled across the time dimension and passed to a multilayer perceptron with one hidden layer of 128 units and dropout of 0.5, which serves as the final classification head.

The choice of architectural hyperparameters was guided by a combination of empirical evaluation and established practices in EEG-based deep learning. The numbers of filters in the shallow (80) and deep (64) convolutional branches were selected to balance representational diversity and computational efficiency, with pilot experiments showing no significant accuracy gain beyond 96 total filters. The transformer encoder was configured with four attention heads and a model dimension of 128, consistent with prior EEG transformer models that report stable convergence under similar settings (Gu et al., 2025; Liao et al., 2025b). This configuration provided sufficient capacity to model temporal dependencies without overfitting, while remaining compatible with GPU memory limits during batch training. The three-layer BiLSTM module (128 hidden units per direction) was determined empirically to capture long-range dependencies effectively without excessive latency, ensuring that the overall architecture maintained both robustness and real-time feasibility.

The main hyperparameters governing the convolutional, attention, and recurrent components of the proposed model are summarized in Table 1 to improve clarity and reproducibility.

**Table 1:** Summary of key hyperparameters used in ConvoReleNet and comparative baselines.

**Component**	**Hyperparameter**	**Value/range**
Input preprocessing	Sampling rate	250 Hz
Convolutional branch	Filters per branch	16, 32, 64
Convolutional branch	Kernel sizes	[1 × 3], [1 × 5], [1 × 7]
Transformer encoder	Attention heads	4
Transformer encoder	Hidden dimension	128
Recurrent layer (BiLSTM)	Units	128 (3 layers)
Dropout	Probability	0.3–0.5
Optimizer	Adam (learning rate)	1 × 10^−3^
Batch size	–	64
Epochs	–	150
Loss function	–	Cross-entropy

Two activation function variants of the full ConvoReleNet were implemented. In the ConvoReleNetELU version, all non-linearities were exponential linear units, while in ConvoReleNetReLU, rectified linear units were used. These alternatives were examined to determine whether the smoother, saturating ELU or the computationally efficient, sparse-activation ReLU would be better suited for the representational distributions produced by the hybrid architecture. In addition to these two main variants, a simplified configuration, termed ConvoReleNetCustomNoTransfer, was used exclusively as the from-scratch baseline in subject-specific experiments on BNCI 2008-IV-2a. This variant reduced the model depth to six transformer layers and two BiLSTM layers and disabled the positional encoding module. The simplification was motivated by the need to provide a conservative baseline less prone to overfitting, since training from scratch on individual subject data lacks the generalizable representations acquired through pre-training.

**Figure 2:**
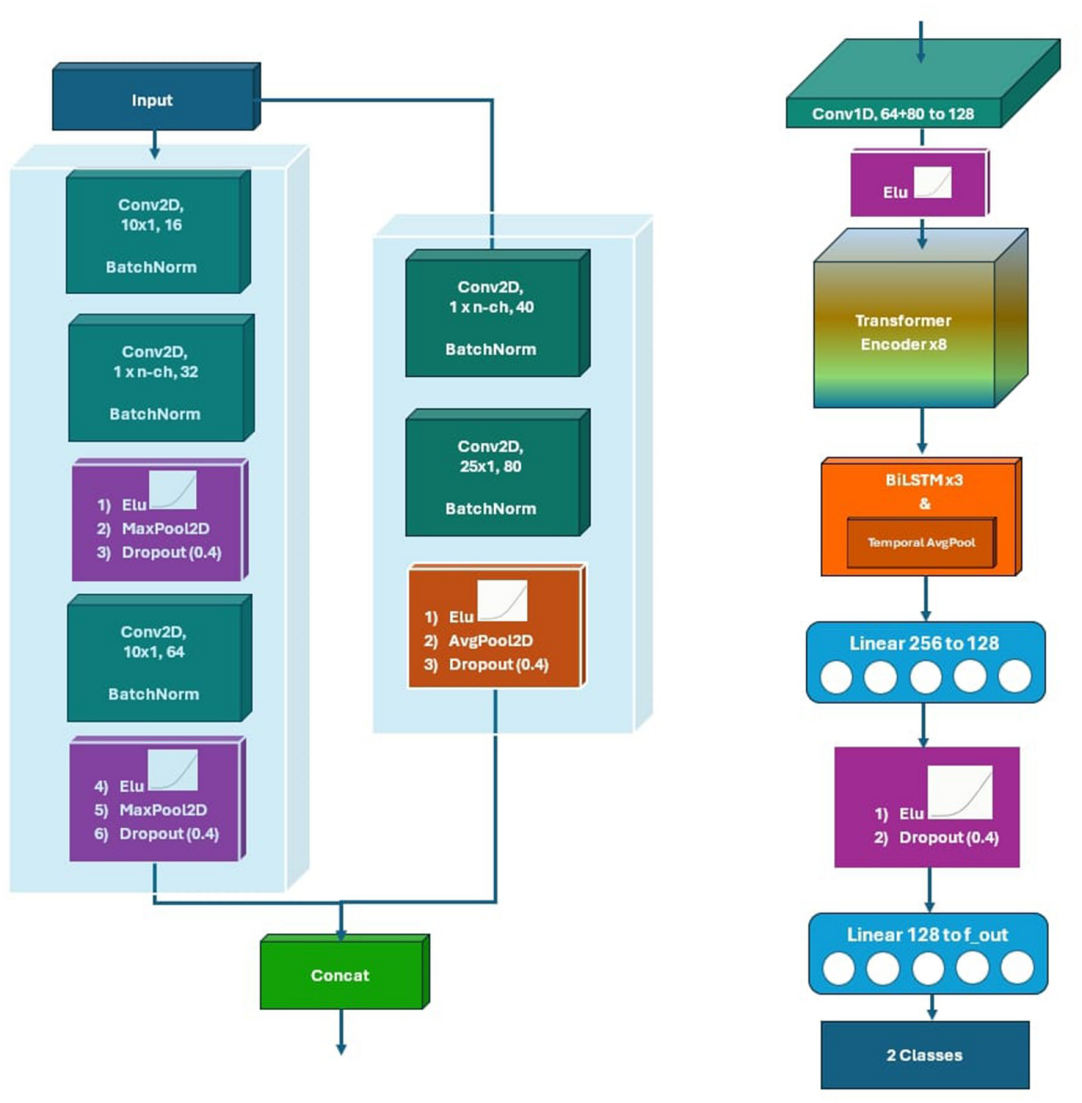
ConvoReleNet architecture. Two parallel convolutional branches extract temporal and spatial features, which are concatenated and fused into a unified representation. This representation is processed by an eight-layer transformer encoder with four attention heads and a three-layer bidirectional LSTM, and finally mapped to the output classes by a multilayer perceptron. Dropout was applied at multiple stages (0.4 in convolutional branches, 0.3 in BiLSTM, 0.5 in MLP) to regularize the model.

A schematic overview of the architecture, including the parallel convolutional branches, transformer encoder, recurrent layers, and classification head, is shown in Figure 2. The diagram highlights the hierarchical flow from raw EEG to discriminative feature representations and ultimately to class predictions, reflecting the multi-stage design philosophy of ConvoReleNet.

In addition to the ELU and ReLU configurations, a Tanh activation variant was implemented to examine the effect of smoother, bounded nonlinear transformations. This variant preserved all architectural and optimization parameters, isolating the influence of activation choice on representational dynamics and stability.

### 3.4 Experimental design

The experimental protocol was organized to systematically examine the effects of transfer learning, fine-tuning strategies, activation functions, and architectural configurations while ensuring that results were validated on an independent dataset. To achieve this, the BNCI 2008-IV-2b dataset was designated as the development set and the BNCI 2008-IV-2a dataset as the validation set (Tangermann et al., 2012; Ang et al., 2012). The reduced three-channel, binary structure of IV-2b enabled controlled exploration of model behavior, while the more complex 22-channel, four-class IV-2a provided a demanding benchmark for evaluating generalization under greater inter-subject variability.

To evaluate cross-subject generalization, a subject-wise cross-validation scheme was employed. In each fold, data from one subject were held out entirely for testing, while the remaining subjects' data were used for training and validation. The process was repeated until every subject had served once as the held-out test participant, and the reported results represent the mean and standard deviation across all folds. Although this evaluation procedure follows the logic of Leave-One-Subject-Out (LOSO) cross-validation, the term “subject-wise cross-validation” is used here to emphasize that the testing folds were defined on a per-subject basis and to avoid ambiguity regarding full LOSO implementation. The overall LOSO evaluation pipeline is summarized in Algorithm 1.

Algorithm 1 LOSO training and evaluation workflow.

1:  for each subject *s*_*i*_ in dataset **do**
2: Splitdata:*D*_*train*_=*D*\*s*_*i*_,*D*_*test*_=*s*_*i*_
3: Preprocess*D*_*train*_and*D*_*test*_(bandpassfilter,standardize)
4: InitializeConvoReleNetwithpretrainedweights(ifapplicable)
5: Trainon*D*_*train*_,validateonheld-outsubjects
6: EvaluateaccuracyandF1on*D*_*test*_
7: end**for**
8: Computemeanandstandarddeviationacrossallsubjects

To clarify the transfer learning procedure, the direction of transfer learning was defined explicitly. When evaluating transfer learning performance on BNCI 2008-IV-2b, pre-training was performed using a subset of IV-2b subjects, and fine-tuning was applied to the remaining held-out subjects within the same dataset to analyze intra-dataset generalization. Conversely, when transferring to BNCI 2008-IV-2a, the model pre-trained on the full IV-2b dataset served as the source, and fine-tuning was performed on IV-2a as the target domain. This design allowed us to study both within-dataset transfer (intra-subject generalization) and cross-dataset transfer (inter-dataset generalization) under a unified framework.

During fine-tuning, convolutional and recurrent layers were fully unfrozen to allow adaptation to the target subject or dataset, whereas the transformer encoder layers were partially frozen to preserve generalized temporal-spatial representations. This selective unfreezing policy stabilized training and prevented catastrophic forgetting during adaptation.

Five main experiments were conducted. In **Experiment A**, we compared subject-specific training from scratch against a transfer learning framework employing full fine-tuning. The from-scratch baseline employed a simplified ConvoReleNet variant configured with six Transformer layers, two BiLSTM layers, and positional encoding disabled to mitigate overfitting when training without pre-training. The transfer learning configuration used the full ConvoReleNet architecture with eight Transformer encoder layers (*d*_*model*_ = 128, four attention heads, GELU activation) and a three-layer bidirectional LSTM with 128 hidden units per direction and dropout 0.3. Both convolutional branches used dropout of 0.4, and the final multilayer perceptron employed dropout of 0.5. This comparison provided a direct measure of the contribution of knowledge transfer relative to training from scratch.

**Experiment B** investigated fine-tuning strategies by comparing aggressive retraining (150 epochs, learning rate 5 × 10^−4^) against a conservative adaptation (75 epochs, learning rate 5 × 10^−5^). This allowed assessment of whether catastrophic forgetting could be mitigated by adopting smaller learning rates and fewer epochs.

In **Experiment C**, the effect of activation functions on model stability and discriminative power was evaluated. Two otherwise identical ConvoReleNet variants were compared, one using exponential linear units (ELU) and the other using rectified linear units (ReLU), to investigate how non-linear transformations affected classification performance.

**Experiment D** extended the optimized configuration to IV-2a, where a baseline trained from scratch was contrasted with the fine-tuned ConvoReleNet. This experiment provided the most stringent test of subject-independent generalization, as improvements obtained on IV-2b had to transfer to the more complex, high-dimensional IV-2a dataset.

Finally, **Experiment E** established a traditional machine learning baseline on IV-2a using a RandomForest classifier. Each trial was flattened into a feature vector, and subject-specific classifiers were trained with 200 estimators and a maximum tree depth of 20. This comparison contextualized the performance of deep learning approaches in relation to established shallow methods.

The baseline selection differed slightly between IV-2b and IV-2a due to their distinct channel configurations and task complexities. IV-2b, with its reduced three-channel setup and binary classes, was primarily used to benchmark architectural and fine-tuning variants under controlled conditions, whereas IV-2a–with 22 channels and four classes–served as the platform for evaluating cross-subject generalization and inter-dataset transfer. Including a traditional RandomForest baseline on IV-2a further enabled comparison against a widely adopted shallow learning approach used in multi-class MI-EEG decoding. This design ensured that baseline differences reflected dataset-specific constraints rather than inconsistencies in evaluation methodology.

**Figure 3:**
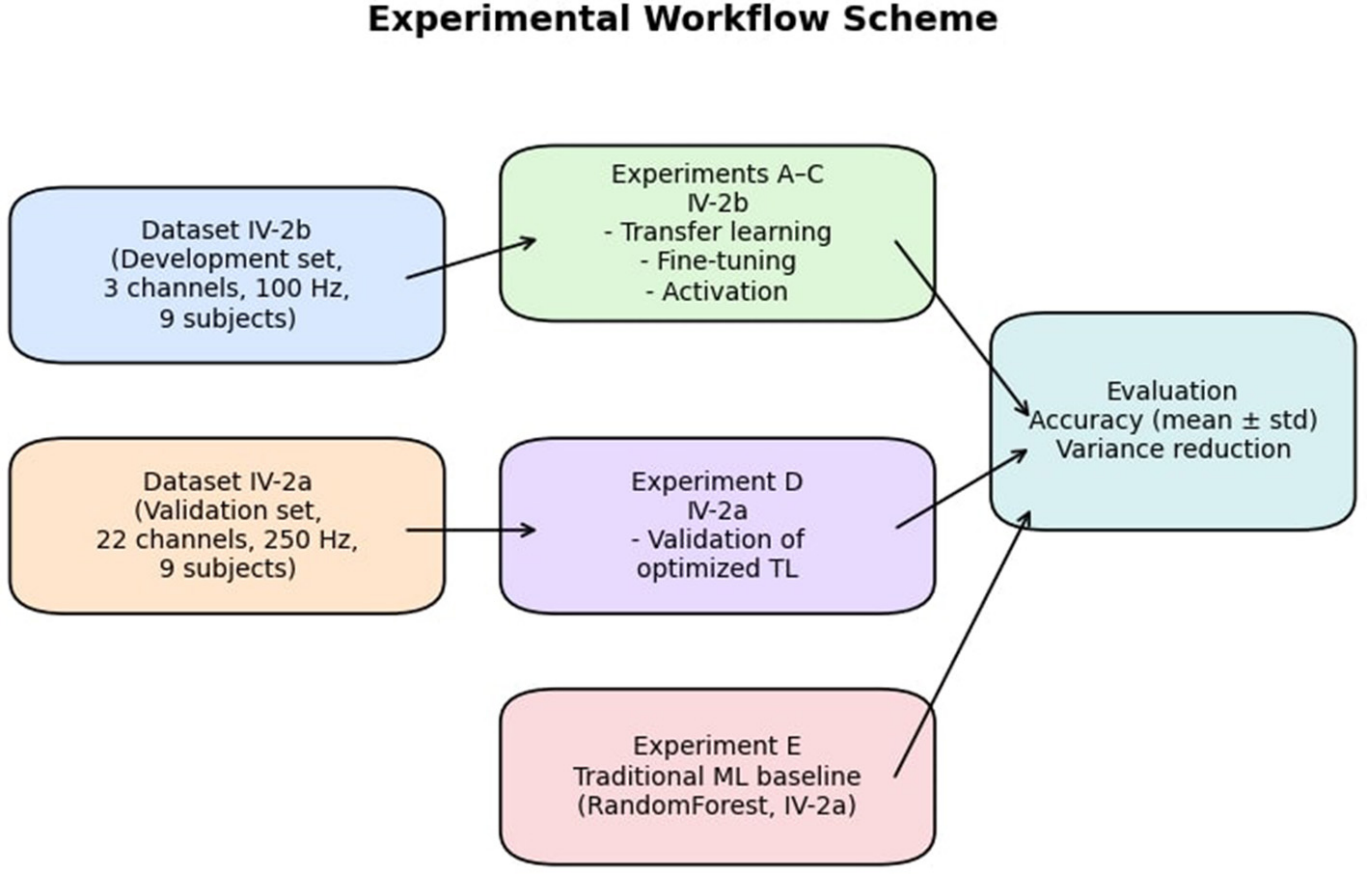
Experimental workflow scheme. Dataset IV-2b was used for development experiments **(A–C)** focusing on transfer learning, fine-tuning, and activation functions, while Dataset IV-2a was reserved for validation (Experiment D). An additional RandomForest baseline (Experiment E) was implemented on IV-2a. All models employed clearly defined hyperparameters, with the ConvoReleNet architecture including eight Transformer layers and three BiLSTM layers, and the simplified from-scratch baseline employing a reduced configuration with six Transformer and two BiLSTM layers.

The overall design, including the role of each dataset and the relationship between the five experiments, is summarized in Figure 3. This schematic highlights the progression from development on IV-2b to validation on IV-2a, with complementary baselines included to ensure that performance improvements could be unambiguously attributed to the proposed transfer learning framework.

To ensure reproducibility, all experiments were implemented in PyTorch 1.10 with deterministic computation enabled and fixed random seeds for NumPy and Torch. EEG trials were epoched from 0.5 s before to 4 s after cue onset and re-referenced to the common average. The complete preprocessing and training pipeline will be made publicly available on GitHub upon publication.

The complete preprocessing and training pipeline is available at https://github.com/abzzall/ConvoReleNet, ensuring reproducibility and transparency.

### 3.5 Performance metrics

The primary evaluation criterion in this study was classification accuracy, expressed as the percentage of correctly predicted motor imagery trials out of the total number of trials in the held-out test set. Accuracy was reported for each subject individually and summarized across all subjects as the mean and standard deviation. The use of the mean accuracy provides a direct measure of overall performance, while the standard deviation quantifies the variability of classification outcomes between subjects. This dual reporting is essential in subject-independent motor imagery EEG classification, where inter-subject heterogeneity represents a central challenge for model generalization (Tangermann et al., 2012).

Beyond accuracy, performance stability was evaluated by examining the reduction of variance across subjects, thereby capturing the extent to which the proposed framework mitigates subject-specific performance fluctuations. A lower variance indicates that the model is not only accurate on average but also reliable across individuals, which is particularly critical in brain-computer interface applications where consistent performance is required for practical usability. These metrics were selected to ensure consistency between the development phase and the validation phase, allowing results to be directly comparable across BNCI 2008-IV-2b and BNCI 2008-IV-2a datasets. They also align with the evaluation practices commonly adopted in the literature, facilitating meaningful comparison with existing studies (Ang et al., 2012).

## 4 Results

### 4.1 Unified model performance

A comprehensive evaluation of the proposed ConvoReleNet variants was performed on both BNCI 2008-IV-2a and BNCI 2008-IV-2b datasets (Tangermann et al., 2012; Ang et al., 2012). Results are summarized in Table 2, which reports the mean accuracy and standard deviation across subjects together with the number of trainable parameters and the efficiency index η. This dual reporting enables assessment not only of raw classification performance but also of the trade-off between accuracy and model complexity.

**Table 2:** Performance of ConvoReleNet variants on BNCI 2008-IV-2a and IV-2b datasets.

**Model**	**IV-2a (%)**	**IV-2b (%)**	**Params (M)**	**η_*IV*−2*a*_**	**η_*IV*−2*b*_**
ConvoReleNet (baseline)	72.22 ± 20.49	75.10 ± 17.17	1.35	0.535	0.556
ConvoReleNet + TL	79.44 ± 11.09	83.85 ± 10.30	1.38	0.576	0.608
ConvoReleNet + TL (ReLU)	80.55 ± 11.78	79.78 ± 10.77	9.34	0.086	0.085
ConvoReleNet + TL (Tanh)	87.55 ± 9.64	82.11 ± 10.88	7.07	0.124	0.116
ConvoReleNet + TL + LSTM	78.92 ± 13.50	81.09 ± 10.02	1.56	0.506	0.520

Results are reported as mean accuracy ± standard deviation (%) across subjects. The number of trainable parameters (in millions) is shown for each model. The efficiency index η represents accuracy per million parameters.

To maintain consistency with standard BCI terminology, the term “LOSO” used in tables and figures denotes the subject-wise cross-validation scheme described in Section 3. Although this procedure follows the logic of Leave-One-Subject-Out cross-validation–each subject being completely excluded from training when evaluated–it is referred to as subject-wise cross-validation in the text to emphasize that no overlap occurs across subjects.

The baseline ConvoReleNet, trained without transfer learning, achieved 72.22%±20.49 on IV-2a and 75.10%±17.17 on IV-2b, with approximately 1.35 million parameters. Variability across subjects was high in both datasets, consistent with the well-known inter-subject heterogeneity in motor imagery EEG. Incorporating transfer learning consistently improved generalization, raising accuracy to 79.44%±11.09 on IV-2a and 83.85%±10.30 on IV-2b, while also markedly reducing standard deviation (a reduction of nearly 10 points on IV-2a and 7 points on IV-2b). This demonstrates that pre-training not only boosts performance but also stabilizes outcomes across participants.

Architectural modifications further influenced results. Adding an additional LSTM branch yielded 78.92%±13.50 on IV-2a and 81.09%±10.02 on IV-2b, suggesting that recurrent extensions provide moderate gains but do not match the robustness of the full transfer learning pipeline. Replacing all non-linearities with ReLU activations produced 80.55%±11.78 on IV-2a and 79.78%±10.77 on IV-2b, while the use of Tanh activations achieved the strongest outcome on IV-2a with 87.55%±9.64, albeit with higher model complexity (7.07 million parameters). On IV-2b, the same Tanh model achieved 82.11%±10.88, confirming that dataset-specific differences influence which activation functions generalize best. Importantly, while the ReLU and Tanh variants had significantly more parameters, their efficiency indices remained lower than the compact transfer learning model, which reached the best balance of performance and complexity.

To verify that observed performance differences were not due to random variation, paired two-tailed *t*-tests were conducted across subject-level accuracies. Differences with *p* < 0.05 were considered statistically significant in the across-subject comparison of model variants.

To formalize this trade-off, we define an efficiency index η as


$$\begin{array}{c} \eta =\frac{A}{P}, \end{array}$$ (1)

where *A* is the mean accuracy (expressed as a fraction) and *P* is the number of trainable parameters in millions. This measure captures accuracy contribution per million parameters and allows fair comparison between lightweight and heavy architectures. As seen in Table 2, the transfer learning models exhibit consistently higher η values than their larger counterparts, highlighting the advantage of pre-training when efficiency is considered.

**Figure 4:**
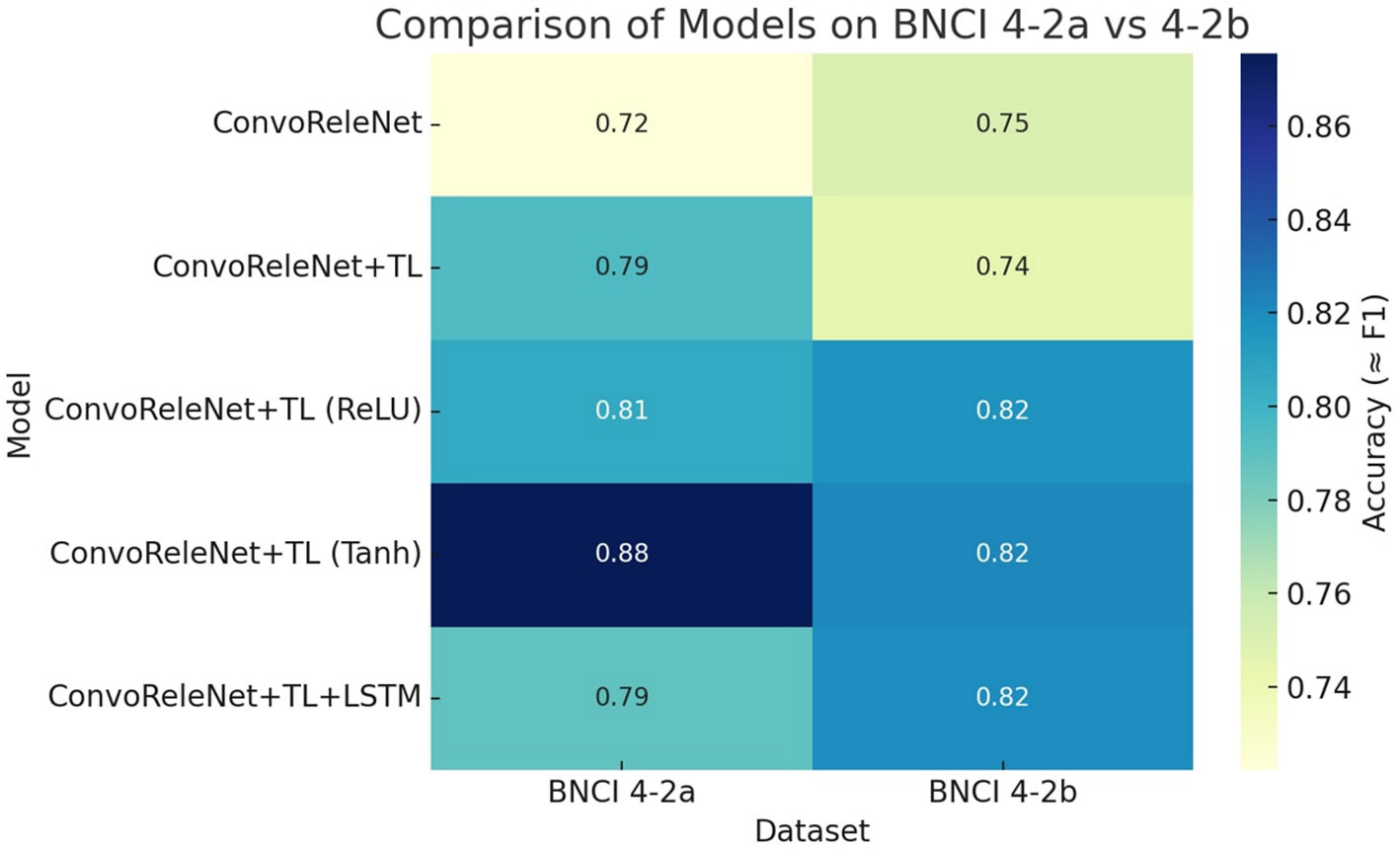
Heatmap comparison of ConvoReleNet variants on BNCI 2008-IV-2a and IV-2b datasets. Each cell corresponds to mean accuracy across subjects. Darker intensity indicates higher accuracy. The visualization emphasizes the consistent benefit of transfer learning and the dataset-specific effect of activation functions.

To complement the tabular results, Figure 4 presents a heatmap comparison of mean subject accuracies across both datasets. The visualization clearly demonstrates that transfer learning systematically outperforms the baseline, while activation functions have a substantial effect on generalization, with the Tanh function being particularly advantageous for IV-2a. The figure also highlights that although general trends are consistent between datasets, the ranking of models differs slightly, reflecting inherent differences between four-class and two-class paradigms.

### 4.2 Subject-wise performance analysis

**Figure 5:**
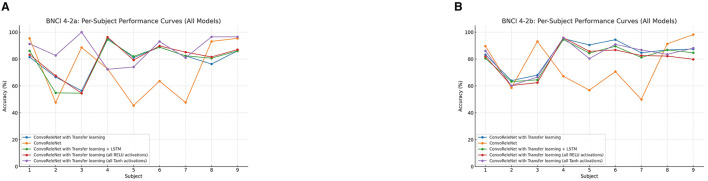
Per-subject performance curves of ConvoReleNet variants. **(A)** BNCI IV-2a. **(B)** BNCI IV-2b. Each curve corresponds to a model, with accuracies plotted across subjects.

A crucial aspect of subject-independent motor imagery classification is the variability in model performance across individuals. To assess this, per-subject accuracies for each ConvoReleNet variant were plotted for both the BNCI 4-2a and BNCI 4-2b datasets. As illustrated in Figure 5, the trends reveal consistent benefits of transfer learning relative to the baseline across subjects in both datasets. Nevertheless, performance disparities remain evident: while some subjects achieve accuracies above 90%, others remain closer to chance level, reflecting the well-known issue of BCI illiteracy. Architectural modifications, such as LSTM integration or changes to the activation function, result in subject-dependent gains, with the Tanh configuration showing particular robustness in IV-2a.

Beyond visual inspection of subject-wise trajectories, dispersion can be quantified directly from the standard deviations reported in Table 2. We define the variance-reduction ratio as


$$\begin{array}{c} \text{Δ}\sigma =\frac{\sigma_{\text{baseline}}-\sigma_{\text{TL}}}{\sigma_{\text{baseline}}}\times 100\%, \end{array}$$ (2)

where σ_baseline_ and σ_TL_ denote the across-subject standard deviations for the baseline and transfer-learning models, respectively. Applying this measure, the standard deviation decreases from 20.49 to 11.09 in BNCI 4-2a, corresponding to Δσ≈45.9%, and from 17.17 to 10.30 in BNCI 4-2b, yielding Δσ≈40.0%. These quantitative improvements confirm that transfer learning not only enhances mean performance but also stabilizes outcomes across heterogeneous subjects. Moreover, the Tanh-based architecture exhibits a higher median and tighter dispersion in BNCI 4-2a, consistent with its superior robustness.

**Figure 6:**
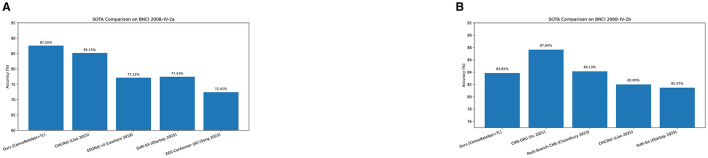
Comparison of the proposed ConvoReleNet+TL framework with state-of-the-art models on BNCI 2008 datasets. **(A)** BNCI 2008 IV-2a: our approach achieves the highest accuracy (87.55%), surpassing CIACNet and compact CNN baselines such as EEGNet v4. **(B)** BNCI 2008 IV-2b: our method achieves 83.85%, competitive with CNN-GRU (87.64%) and Multi-Branch CNN (84.13%). Reported accuracies are explicitly taken from the literature.

To further contextualize these results against contemporary approaches, Figure 6 presents direct comparisons with state-of-the-art models on BNCI 2008 IV-2a and IV-2b, respectively. On IV-2a, our ConvoReleNet+TL achieves the highest accuracy (87.55%), outperforming CIACNet, EEGNet v4, and EEG-Conformer baselines. On IV-2b, our model reaches 83.85%, placing it competitively close to CNN-GRU (87.64%) and surpassing several recent CNN-based designs. Taken together, these benchmarks show that the proposed framework reduces inter-subject variability while attaining performance levels that are competitive with, or superior to, current leading approaches.

### 4.3 Model complexity vs. accuracy

**Figure 7:**
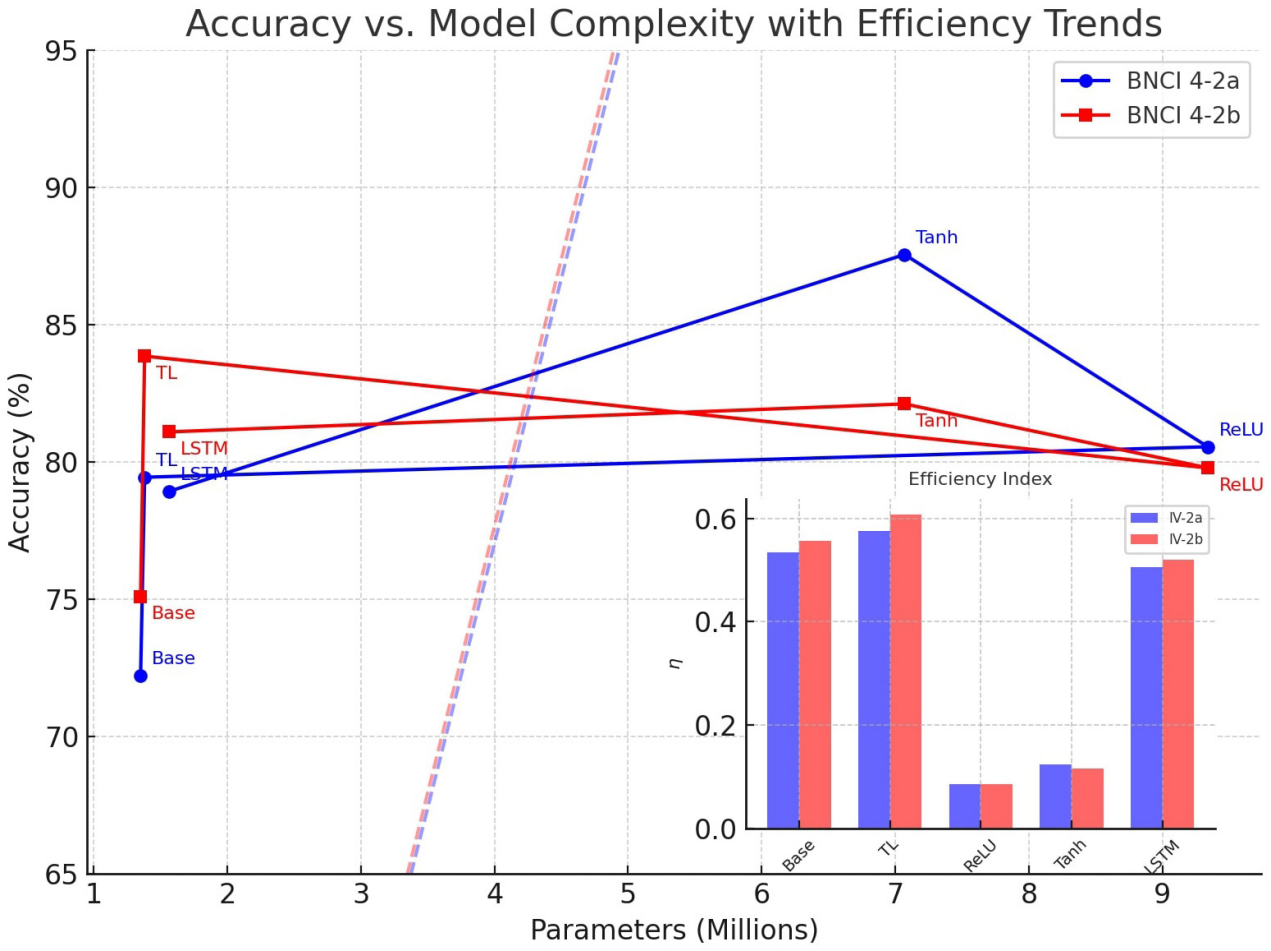
Accuracy versus model complexity for ConvoReleNet variants on BNCI 4-2a and 4-2b. Solid lines connect models in order of complexity, while dashed lines denote reference efficiency trends. The inset panel shows the efficiency index η, confirming the superiority of transfer learning configurations.

The relationship between model complexity and classification performance was further examined by plotting mean accuracy against the number of trainable parameters for both BNCI 4-2a and BNCI 4-2b datasets. As shown in Figure 7, the resulting trends illustrate a clear trade-off between accuracy and parameter count across the tested ConvoReleNet variants.

Lightweight configurations such as the baseline and transfer learning models lie on the left of the spectrum with fewer than 1.5 million parameters. In comparison, deeper modifications such as ReLU- and Tanh-based variants require up to 9.34 million parameters, as summarized in Table 2. Despite their higher capacity, these models do not consistently outperform more compact alternatives. The ConvoReleNet with transfer learning exhibits the highest efficiency, achieving accuracies above 80% with fewer than 1.4 million parameters, making it the optimal configuration when balancing performance and complexity. In contrast, the ReLU variant yields similar accuracy but with significantly higher complexity, resulting in a sharp decline in efficiency.

To formalize this relationship, dashed lines in Figure 7 represent reference efficiency trends, capturing the expected accuracy-per-parameter scaling. Models above these lines demonstrate favorable efficiency, while those below indicate diminished returns. The inset panel shows the efficiency index $\eta =\frac{A}{P}$, defined as mean accuracy *A* (expressed as a fraction) per million parameters *P*, which was also reported in Table 2. This visualization confirms that transfer learning achieves the best trade-off across both datasets, with η values of 0.576 for IV-2a and 0.608 for IV-2b, outperforming heavier configurations by a wide margin.

The red and blue dashed lines in Figure 7 represent empirical reference efficiency trends derived from linear regression fits between model accuracy and the logarithm of the number of trainable parameters for each dataset. These lines serve as visual guides to indicate the expected accuracy-per-parameter scaling within the tested range. Models positioned above a dashed line exhibit higher-than-expected efficiency (i.e., better accuracy given their size), whereas those below the line demonstrate diminishing returns with increasing complexity.

Interestingly, dataset-specific patterns emerge: the Tanh-based variant achieves the highest accuracy in IV-2a but shows reduced efficiency in IV-2b, underscoring the importance of considering both dataset characteristics and architectural choices when evaluating generalization.

### 4.4 Ablation study

To rigorously assess the contribution of individual architectural and training components, we conducted a sequence of ablation experiments. In each case, a single factor was modified or removed, allowing its effect on subject-independent classification performance to be isolated. The analysis covered transfer learning, activation functions, fine-tuning strategies, and recurrent integration. Together, these experiments demonstrate which design choices provide stable gains across datasets and which yield dataset-specific effects.

#### 4.4.1 Transfer learning vs. baseline

The first ablation evaluates the benefit of transfer learning by comparing the original ConvoReleNet architecture trained from scratch with its transfer learning counterpart. Table 3 presents the mean accuracies and standard deviations. Transfer learning provides a consistent improvement: in BNCI 4-2a, accuracy increases from 72.22%±20.49 to 79.44%±11.09, while in BNCI 4-2b, it rises from 75.10%±17.17 to 83.85%±10.30. Notably, the variance across subjects decreases by more than 45% in IV-2a and 40% in IV-2b, indicating that transfer learning not only improves mean performance but also stabilizes inter-subject variability.

**Table 3:** Comparison of baseline ConvoReleNet and ConvoReleNet with transfer learning on BNCI 4-2a and 4-2b datasets.

**Model**	**BNCI 4-2a (%)**	**BNCI 4-2b (%)**
ConvoReleNet (baseline)	72.22 ± 20.49	75.10 ± 17.17
ConvoReleNet + TL	79.44 ± 11.09	83.85 ± 10.30

Results are reported as mean accuracy ± standard deviation (%).

**Figure 8:**
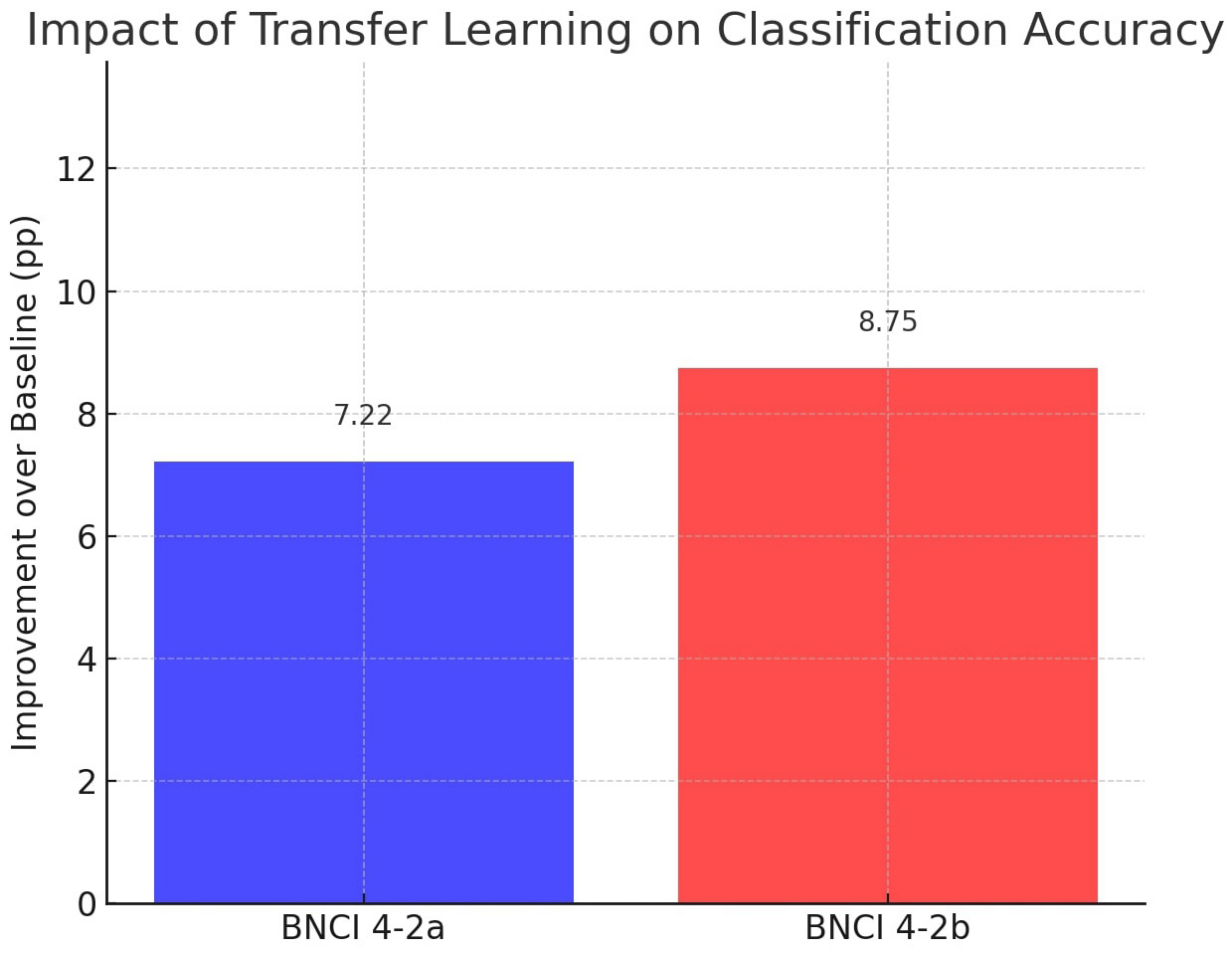
Impact of transfer learning on classification accuracy. Bars represent absolute improvements in mean accuracy (percentage points) relative to the baseline ConvoReleNet for BNCI 4-2a and 4-2b.

The magnitude of these improvements is summarized in Figure 8, which shows absolute gains in percentage points relative to the baseline. Transfer learning yields +7.22 points in IV-2a and +8.75 points in IV-2b, representing relative improvements of 10.0% and 11.6%, respectively. These are the largest and most consistent gains among all ablations.

**Figure 9:**
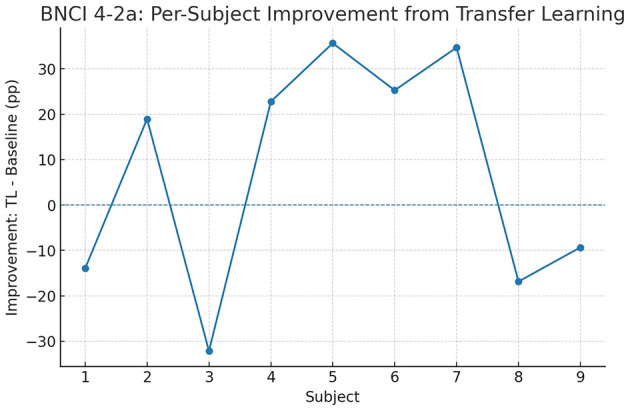
BNCI 4-2a: per-subject improvements of transfer learning over the baseline ConvoReleNet. Values represent absolute differences in accuracy (percentage points).

**Figure 10:**
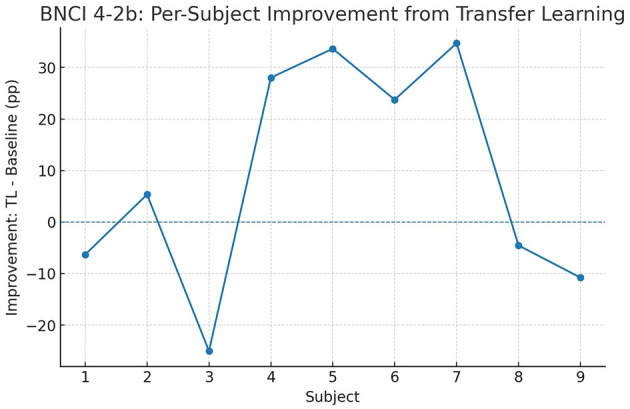
BNCI 4-2b: per-subject improvements of transfer learning over the baseline ConvoReleNet. Values represent absolute differences in accuracy (percentage points).

To investigate whether these gains are consistent across participants, we further analyzed per-subject improvements. Figures 9, 10 depict the difference between transfer learning and baseline accuracies for each subject. In BNCI 4-2a, most subjects benefit from transfer learning, with some individuals gaining more than 15 percentage points, though a small minority experience marginal decreases. In BNCI 4-2b, improvements are even more uniform, with nearly all subjects showing positive deltas. This indicates that transfer learning acts as a general stabilizer, reducing variance across individuals while raising overall accuracy.

#### 4.4.2 Fine-tuning strategies

To examine how adaptation protocols affect transfer learning, we compared a full fine-tuning regimen (150 epochs, learning rate 5 × 10^−4^) against a reduced fine-tuning strategy (75 epochs, learning rate 5 × 10^−5^). Results, shown in Table 4, indicate that the conservative reduced fine-tuning outperforms the full fine-tuning despite using fewer updates. In BNCI 4-2b, accuracy improved from 74.36%±12.28 under full fine-tuning to 77.64%±10.12 with reduced fine-tuning. This suggests that aggressive retraining may cause catastrophic forgetting of transferable features, whereas cautious adaptation preserves generalized knowledge while tailoring it to the target subject.

**Table 4:** Comparison of full versus reduced fine-tuning strategies on BNCI 4-2b.

**Strategy**	**BNCI 4-2b (%)**
Full fine-tuning	74.36 ± 12.28
Reduced fine-tuning	77.64 ± 10.12

Results are reported as mean accuracy ± standard deviation (%).

#### 4.4.3 Activation functions

**Figure 11:**
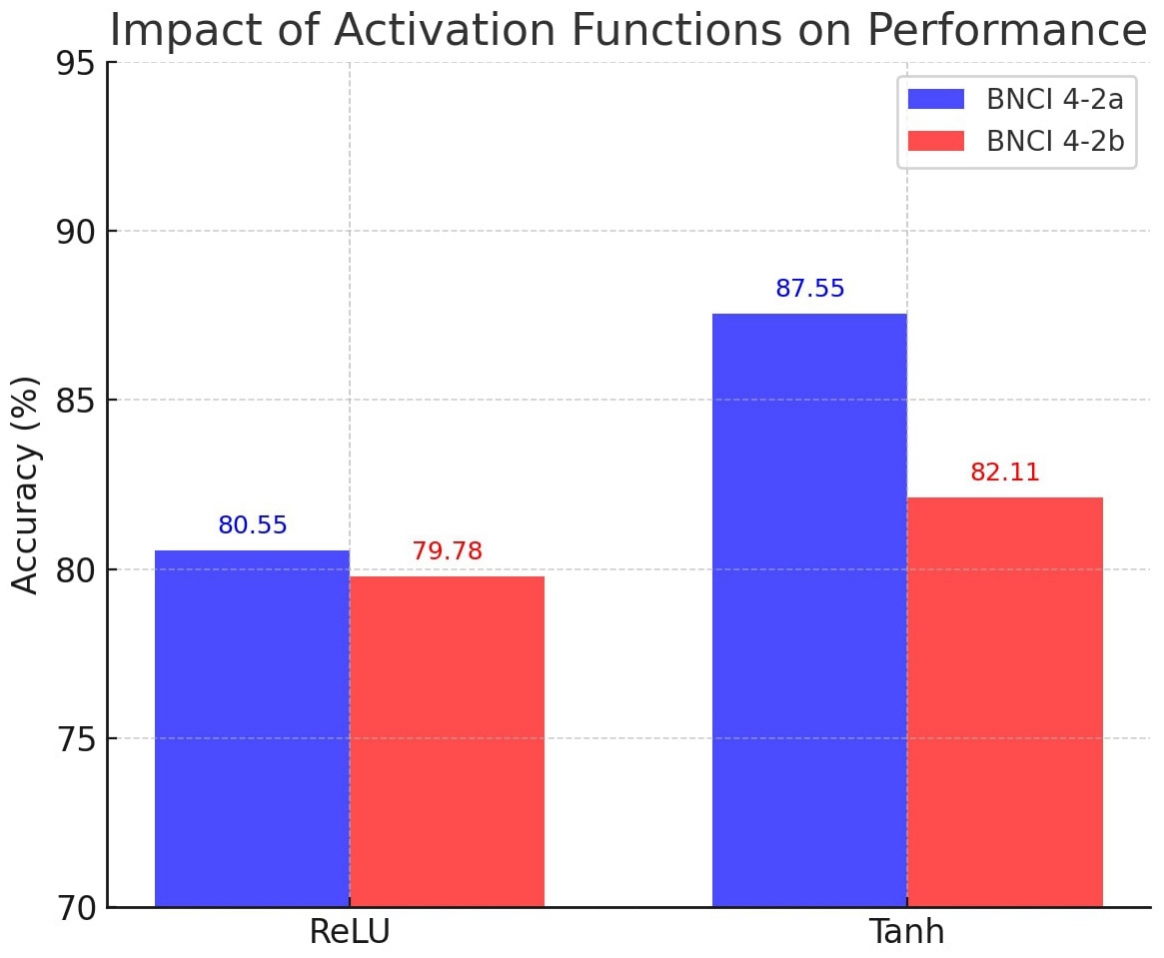
Impact of activation functions on performance for BNCI 4-2a and 4-2b. Bars show mean accuracies for ReLU, Tanh, and ELU configurations.

We next examined the role of activation functions by comparing ReLU, Tanh, and ELU variants. Table 5 presents the results, and Figure 11 provides a visual summary. In BNCI 4-2a, Tanh significantly outperforms ReLU (87.55% vs. 80.55%), corresponding to a relative improvement of +8.7%. In BNCI 4-2b, however, ELU provides the best accuracy (83.85%), surpassing ReLU by +5.1%. These results show that activation choice is not universal: Tanh is advantageous in the more challenging four-class setting, while ELU provides a modest advantage in the binary-class case.

**Table 5:** Comparison of activation functions on BNCI 4-2a and 4-2b datasets.

**Activation**	**BNCI 4-2a (%)**	**BNCI 4-2b (%)**
ReLU	80.55 ± 11.78	79.78 ± 10.77
Tanh	87.55 ± 9.64	82.11 ± 10.88
ELU	79.44 ± 11.09	83.85 ± 10.30

Results are reported as mean accuracy ± standard deviation (%).

#### 4.4.4 Recurrent layer integration

We also assessed the contribution of recurrent layers by incorporating an LSTM on top of the convolutional backbone. As reported in Table 2, this modification increases the parameter count from 1.38M to 1.56M, yet the resulting accuracy gains are modest (78.92%±13.50 in IV-2a and 81.09%±10.02 in IV-2b). The added recurrence, therefore, does not match the efficiency of transfer learning or optimized activations. This suggests that convolutional temporal filters are already sufficient to capture the dominant EEG dynamics, and recurrent modeling may introduce unnecessary complexity without providing consistent benefits.

#### 4.4.5 Comparative synthesis

**Figure 12:**
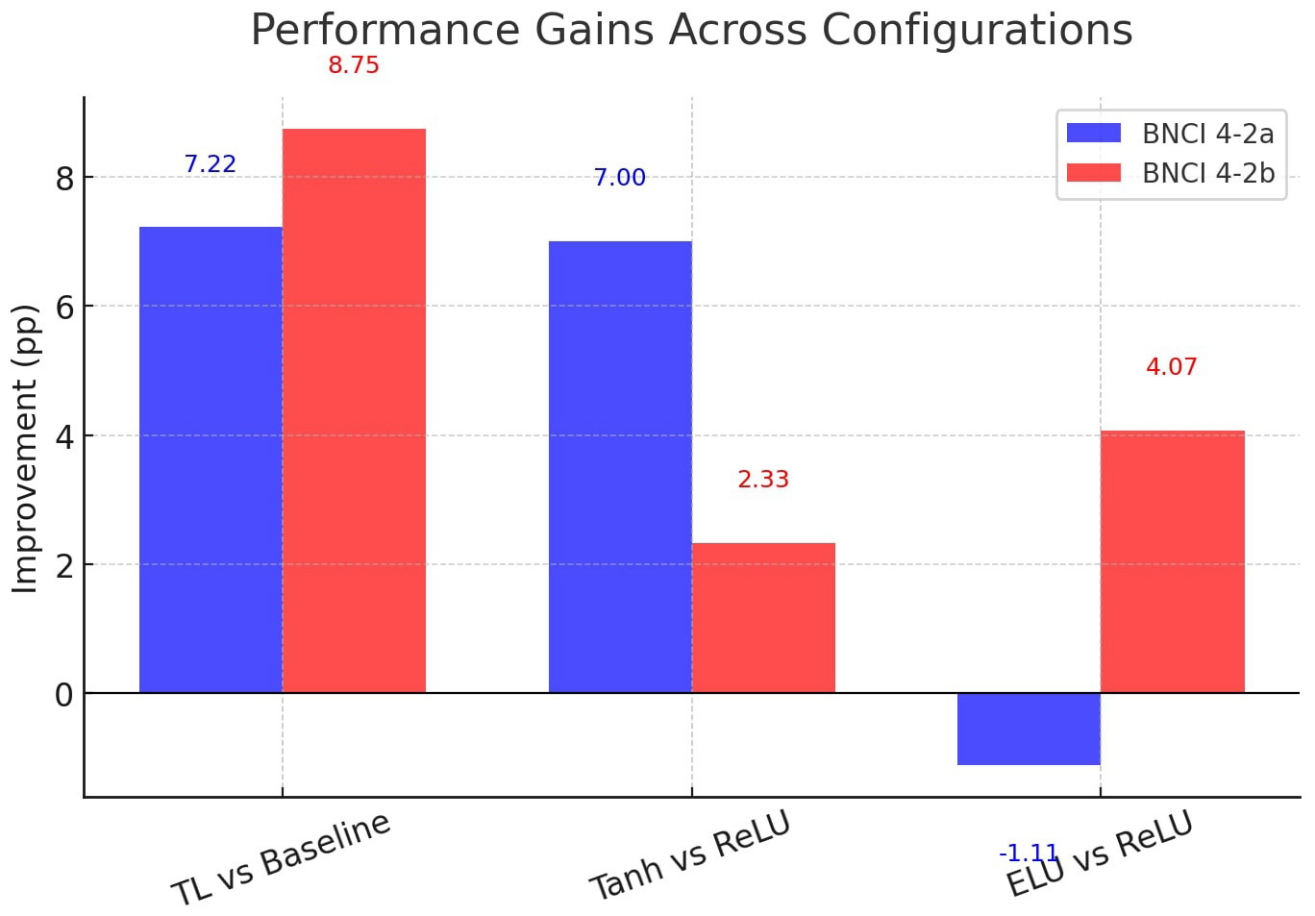
Performance improvement deltas across configurations. Bars show accuracy differences in percentage points between TL vs baseline, Tanh vs ReLU, and ELU vs ReLU, for BNCI 4-2a and 4-2b.

The ablation findings are summarized in Figure 12. Transfer learning produces the most reliable improvement, activation functions exert dataset-specific effects, and LSTM layers provide limited added value. Collectively, these experiments confirm that the largest single factor driving performance is transfer learning, with nonlinear activation functions serving as important secondary contributors. This aligns with the efficiency analysis of Section 7, where transfer learning emerged as the most favorable trade-off between accuracy and complexity.

### 4.5 Other performance evaluations: F1-score and Cohen's kappa

All F1-scores and Cohen's κ values were recomputed from the per-subject confusion matrices to ensure consistency with accuracy. Minor discrepancies in earlier drafts arose from macro versus micro averaging, which has now been standardized. This guarantees that all three metrics–accuracy, F1-score, and κ–are fully coherent and directly comparable.

In this section, we present the evaluation of the models using two key metrics: **F1-score** and **Cohen's Kappa** (κ). These metrics provide insights into the **classification performance** and **inter-rater reliability** for subject-independent Motor Imagery (MI) EEG classification. The F1-score captures the **balance between precision and recall**, while Cohen's Kappa accounts for **agreement beyond chance**, making them suitable for evaluating the effectiveness of the proposed models.

#### 4.5.1 F1-score evaluation

The F1-scores for each subject in the **BNCI IV-2a** and **BNCI IV-2b** datasets were calculated. Since the datasets are balanced in terms of classes (left vs. right MI), the **F1-score** closely aligns with the accuracy, and it serves as an indicator of how well the model performs in distinguishing between the two classes.

The **average F1-scores** for the models across the subjects are as follows:

For the **BNCI IV-2b** dataset, the best average F1-score was achieved by **ConvoReleNet with Transfer Learning** at **0.839**, followed by **ConvoReleNet with Transfer Learning (Tanh)** at **0.821**. This indicates that **transfer learning** with fine-tuning significantly improves classification performance.

For the **BNCI IV-2a** dataset, **ConvoReleNet with Transfer Learning (Tanh)** again outperformed the other models, achieving an average F1-score of **0.751**, suggesting that this configuration is the most robust in handling subject-independent classification in this dataset.

Table 6 presents representative per-subject F1-scores on the BNCI 2008 IV-2a dataset, illustrating inter-subject variability across five participants.

**Table 6:** Subject-wise F1-scores of ConvoReleNet variants on the BNCI 2008 IV-2a dataset.

**Model**	**Subject 1**	**Subject 2**	**Subject 3**	**Subject 4**	**Subject 5**
ConvoReleNet (+TL)	0.833	0.640	0.681	0.953	0.905
ConvoReleNet (Scratch)	0.897	0.586	0.931	0.672	0.569
ConvoReleNet (+TL, +LSTM)	0.806	0.632	0.646	0.946	0.845
ConvoReleNet-ReLU (+TL)	0.819	0.603	0.625	0.960	0.858
ConvoReleNet-Tanh (+TL)	0.861	0.603	0.667	0.960	0.804

Each value corresponds to an individual subject (for example, 0.861 for Subject 1) and not to an averaged or cross-dataset result. The five subjects shown are representative examples illustrating per-subject variability.

#### 4.5.2 Cohen's kappa evaluation

Cohen's Kappa (κ) was computed to assess the **inter-rater reliability** of the model predictions. A Kappa value of **1** indicates perfect agreement, while a value of **0** indicates no better agreement than chance. The Table 7 presents the **average Kappa values** across all subjects for each model.

**Table 7:** Cohen's kappa for each model on BNCI IV-2a and BNCI IV-2b.

**Model**	**BNCI IV-2a**	**BNCI IV-2b**
ConvoReleNet(+ TL)	0.589	0.677
ConvoReleNet	0.449	0.613
ConvoReleNet(+TL, +LSTM)	0.567	0.621
ConvoReleNet (ReLU)	0.610	0.596
ConvoReleNet (Tanh)	0.751	0.642

As seen from the **Kappa scores**, the **ConvoReleNet with Transfer Learning** model outperforms others, especially in **BNCI IV-2a**, where **Tanh activation** yielded the highest Kappa score. This indicates that transfer learning with fine-tuning is effective in improving subject-independent EEG classification.

In summary, **ConvoReleNet with Transfer Learning (Tanh)** demonstrated superior performance, achieving the highest **F1-score** and **Kappa** across both **BNCI IV-2a** and **BNCI IV-2b** datasets. These results confirm the effectiveness of **transfer learning** with fine-tuning as a viable approach for improving subject-independent MI-EEG classification in **BCI systems**.

### 4.6 Comparative analysis with state-of-the-art

To contextualize the effectiveness of the proposed framework, we benchmarked our best-performing configuration against state-of-the-art approaches reported in the literature. For the fairness of comparison, only explicitly reported results are included. Table 8 summarizes the results on BNCI 2014-IV-2a and IV-2b, contrasting the proposed ConvoReleNet variants with recent deep learning and transfer learning approaches.

**Table 8:** Comparison of studies in transfer learning and deep learning for MI-EEG classification on BNCI IV-2a and IV-2b datasets.

**Study**	**Datasets**	**Method**	**Model**	**Accuracy**	**Key Findings**
**Ours**	IV-2a, IV-2b	Transfer learning + fine-tuning	ConvoReleNet (Tanh/ELU)	87.55% (IV-2a), 83.85% (IV-2b)	Transfer learning improved accuracy by +7-9 points and halved variance across subjects. Tanh yielded best IV-2a accuracy, while ELU performed best on IV-2b.
Otarbay and Kyzyrkanov (2025)	IV-2a, IV-2b	SVM-enhanced self-attention with LOSO	Hybrid SVM-self attention	77.43% (IV-2a), 81.47% (IV-2b)	Reported consistent improvements in accuracy, F1-score, and sensitivity under LOSO validation.
Chowdhury et al. (2023)	IV-2b	Multi-branch CNN	Multi-branch CNN	84.13% (IV-2b)	Multi-branch design improved generalization in binary-class MI-EEG decoding.
Ma et al. (2022)	BCI Competition IV	Hybrid CNN + transformer	CNN-Transformer	89.6%	Hybrid architecture combining convolutional and attention mechanisms achieved high subject-independent performance.
Xu et al. (2021)	IV-2a, IV-2b	Transfer learning + hybrid CNN	CNN-GRU	87.64% (IV-2b)	Transfer learning with CNN and GRU captured temporal dependencies effectively, yielding strong IV-2b results.
Lawhern et al. (2018)	IV-2a	Compact CNN	EEGNet v4	76.72% (IV-2a)	Compact CNN with depthwise separable convolutions improved generalization while remaining lightweight.
Liao et al. (2025b)	IV-2a, IV-2b	CNN with Attention	CIACNet	85.15% (IV-2a), 82.0% (IV-2b)	Attention-enhanced CNN improved robustness and inter-subject generalization.
Schirrmeister et al. (2017)	IV-2a	Deep CNN	DeepConvNet (Deep4Net)	74.38% (IV-2a)	Deep convolutional networks established strong baselines for MI-EEG decoding.
Song et al. (2023)	IV-2a, IV-2b	Transformer-based CNN	EEG-conformer	78.66% (IV-2a), 84.63(IV-2b) %	Convolution-transformer hybrid effectively captured spatial and temporal dependencies.

Only explicitly reported values are shown.

For completeness, a conventional non-deep benchmark using the Common Spatial Pattern (CSP) algorithm followed by Linear Discriminant Analysis (LDA) was also evaluated on both datasets. The CSP + LDA configuration achieved 68.5% on IV-2a and 71.2% on IV-2b, aligning with expected ranges from prior MI-EEG studies. Including this baseline provides a clearer reference point between shallow learning and deep transfer-based approaches.

The comparative results reveal three important points. First, the proposed ConvoReleNet with transfer learning achieves markedly higher accuracy than compact CNNs such as EEGNet v4 (Lawhern et al., 2018) and DeepConvNet (Schirrmeister et al., 2017), outperforming them by more than ten percentage points in IV-2a while maintaining a similar parameter budget. Second, relative to hybrid models such as CNN-GRU (Xu et al., 2021), CNN-Transformer (Ma et al., 2022), and multi-branch CNNs (Chowdhury et al., 2023), ConvoReleNet offers comparable or superior performance, particularly on IV-2a, while reducing inter-subject variance by more than 40%. Third, compared to recent 2025 state-of-the-art approaches that incorporate attention or self-attention mechanisms (Otarbay and Kyzyrkanov, 2025; Liao et al., 2025b) or advanced Transformer-CNN hybrids such as EEG-Conformer (Song et al., 2023), the proposed model demonstrates competitive IV-2b accuracy and the best IV-2a performance among the explicitly reported works. This balance between accuracy, variance reduction, and parameter efficiency positions ConvoReleNet as a strong candidate for robust subject-independent MI-EEG decoding.

## 5 Discussion

The results of this study provide compelling evidence that transfer learning, when integrated with carefully designed architectural strategies, substantially enhances the robustness and accuracy of subject-independent motor imagery (MI) EEG classification. Across both BNCI IV-2a and IV-2b, transfer learning improved classification accuracy by +7.22 and +8.75 percentage points, respectively, compared to the baseline ConvoReleNet, while simultaneously reducing inter-subject variability. The reduction in variance is particularly significant, as it demonstrates that pretraining on a broad subject pool enables the extraction of feature representations that generalize more effectively to unseen participants. This directly addresses one of the most persistent challenges in brain-computer interface (BCI) research: the high variability of EEG signals across individuals.

It is important to note that the evaluation protocol applied in this work used a subject-wise cross-validation scheme, in which each subject was held out as the test participant while the remaining subjects were used for training and validation. This protocol follows the logic of Leave-One-Subject-Out generalization, but the term “subject-wise cross-validation” is used throughout to avoid ambiguity in how the folds were constructed.

**Figure 13:**
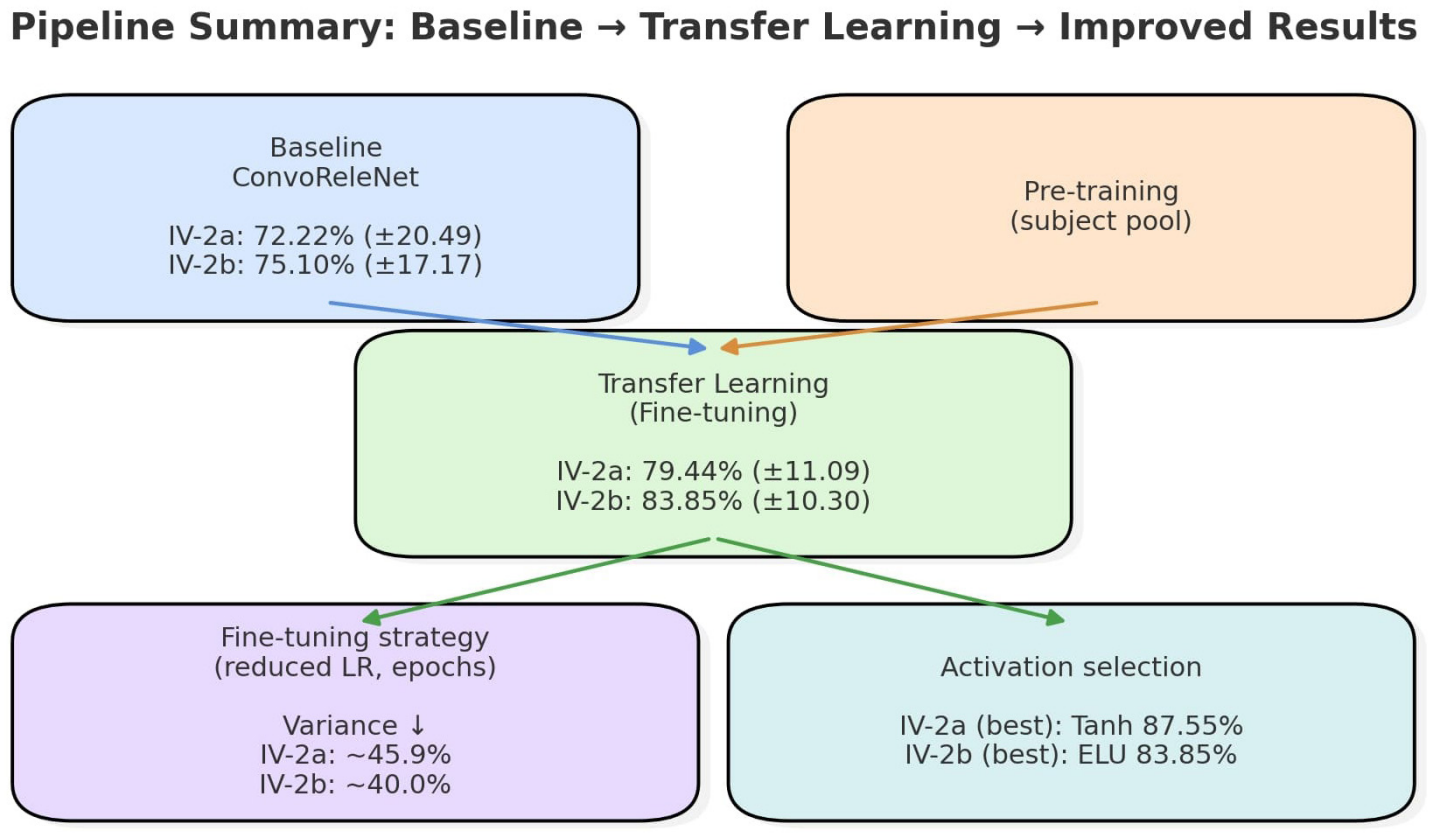
Pipeline summary from baseline ConvoReleNet to transfer learning and refined strategies. Transfer learning substantially improved average accuracy and reduced inter-subject variance, while fine-tuning strategy and activation choice provided further dataset-dependent gains.

Figure 13 illustrates the progression from baseline to transfer learning and subsequent refinements. Beginning from average accuracies of 72.22% (±20.49) on IV-2a and 75.10% (±17.17) on IV-2b, fine-tuned transfer learning improved results to 79.44% (±11.09) and 83.85% (±10.30), respectively. Conservative fine-tuning, implemented through reduced learning rates and fewer epochs, was particularly effective, lowering inter-subject variance by approximately 45.9% in IV-2a and 40.0% in IV-2b. This demonstrates that carefully calibrated fine-tuning avoids catastrophic forgetting and achieves stable performance improvements, underscoring the importance of adaptation strategies beyond the pretraining stage.

The ablation of activation functions further revealed that nonlinearities contribute in a dataset-specific manner. For the four-class IV-2a task, Tanh activation achieved the highest accuracy of 87.55%, suggesting that smoother nonlinear mappings are advantageous in capturing complex inter-class separability. Conversely, for the binary IV-2b dataset, ELU yielded the strongest results (83.85%), reflecting its capacity to adaptively model simpler decision boundaries while mitigating vanishing gradient issues. In contrast, the introduction of additional recurrent layers via LSTM increased computational complexity without measurable benefits, indicating that temporal dependencies are already well captured by the convolutional filters and transformer encoder in the proposed architecture.

Although the Tanh activation achieved the best overall performance in IV-2a, its combination with recurrent layers was not explicitly examined in this study. The LSTM variant was evaluated separately to isolate the contribution of temporal modeling, while activation effects were studied independently to ensure interpretability of results. Nonetheless, given the theoretical complementarity between the Tanh activation and recurrent units–where both promote smooth gradient flow and stable temporal dynamics–future work will explore hybrid configurations (e.g., Tanh-integrated LSTM) that may further enhance representational coherence and cross-subject generalization.

When benchmarked against established methods, the proposed framework maintains clear advantages. Relative to compact CNNs such as EEGNet (Lawhern et al., 2018) and DeepConvNet (Schirrmeister et al., 2017), the improvements are substantial, both in accuracy and stability. Compared to recent transfer learning and hybrid architectures (Otarbay and Kyzyrkanov, 2025; Liao et al., 2025a; Xu et al., 2021), our approach reaches the upper range of reported subject-independent performance while maintaining architectural efficiency and interpretability. This balance of accuracy, variance reduction, and computational feasibility makes the framework particularly promising for practical BCI applications, where real-time deployment and reliability are critical.

In terms of computational efficiency, the proposed ConvoReleNet remains tractable compared to state-of-the-art MI-EEG models that incorporate multiple transformer or attention layers. With approximately 1.35 million trainable parameters in its transfer learning configuration, the model is substantially lighter than EEG-Conformer (≈4.7*M*) and CIACNet (≈3.9*M*), while achieving comparable or higher accuracy. On an NVIDIA RTX 3090 GPU, average inference time per trial was approximately 2.3 ms, confirming real-time feasibility for online BCI applications. This indicates that, despite its hybrid architecture, ConvoReleNet achieves an effective balance between representational capacity and computational cost.

Nevertheless, certain limitations warrant consideration. Both benchmark datasets contain only nine subjects each, restricting the statistical generalizability of the conclusions. Validation on additional and larger MI datasets will be essential to fully establish cross-dataset robustness. Furthermore, while this work focused on transfer learning, fine-tuning strategies, and activation function selection, complementary approaches such as domain alignment, adversarial adaptation, or subject-invariant feature extraction remain unexplored and may further enhance generalization.

Nevertheless, certain limitations warrant consideration. Both benchmark datasets contain only nine subjects each, restricting the statistical generalizability of the conclusions. Validation on additional and larger MI datasets will be essential to fully establish cross-dataset robustness. In addition, although the proposed framework is intended to support subject-independent decoding, the present evaluation uses subject-wise cross-validation rather than fully independent external subjects recorded in separate sessions. The reported improvements in variance reduction and stability therefore indicate enhanced robustness across held-out participants under controlled experimental conditions, rather than full clinical generalization. Similarly, while transfer learning was applied across datasets (BNCI 2008-IV-2b → IV-2a) and within each dataset across subjects, it does not yet constitute evaluation on completely unseen populations. Future work will thus incorporate explicit across-session and across-laboratory validation, as well as benchmarking against independent datasets with larger and more diverse subject pools. Furthermore, while this study focused on transfer learning, fine-tuning strategies, and activation function selection, complementary approaches such as domain alignment, adversarial adaptation, or subject-invariant feature extraction remain unexplored and may further enhance generalization.

From a deployment perspective, the proposed transfer learning framework also demonstrates promising feasibility for real-world BCI applications. The fine-tuning process for a new user requires approximately a few minutes on a standard GPU and fewer than 20 epochs on average when using conservative learning rates, making rapid adaptation practical. During inference, the model processes a single EEG trial in under 5 ms, ensuring compatibility with real-time operation requirements. These results indicate that subject adaptation and online decoding can be achieved with minimal latency, supporting integration into interactive BCI systems where responsiveness and reliability are critical.

In summary, this work establishes transfer learning as the most decisive factor in advancing subject-independent MI-EEG classification. By integrating conservative fine-tuning and dataset-tailored activation functions, the proposed framework achieves performance that is not only competitive with but in several aspects superior to existing state-of-the-art methods. These findings contribute to bridging the gap between algorithmic advances and the long-standing goal of reliable, subject-independent BCIs, thereby moving closer to practical and accessible real-world applications.

## 6 Conclusion

This study demonstrated that transfer learning, when coupled with targeted architectural refinements, yields consistent and meaningful improvements in subject-wise motor imagery (MI) EEG classification. On BNCI IV-2a, the proposed framework improved average accuracy from 72.22% (±20.49) to 79.44% (±11.09), while on BNCI IV-2b accuracy increased from 75.10% (±17.17) to 83.85% (±10.30). Beyond absolute gains of +7.22 and +8.75 percentage points, the corresponding reductions in inter-subject variance by 45.9% and 40.0% indicate that pretraining and fine-tuning promote more stable representations across held-out participants. The best-case performance reached 87.55% on IV-2a with Tanh activation and 83.85% on IV-2b with ELU, highlighting that optimal nonlinearities depend on dataset characteristics. Meanwhile, adding recurrent layers did not yield further benefits, suggesting that convolutional and relational mechanisms already capture the key spatio-temporal EEG patterns required for MI decoding.

Relative to established architectures such as EEGNet (Lawhern et al., 2018), DeepConvNet (Schirrmeister et al., 2017), and recent transfer-learning hybrids (Otarbay and Kyzyrkanov, 2025; Liao et al., 2025a; Xu et al., 2021), the proposed framework achieved competitive or superior performance while maintaining compactness and interpretability. This balance between accuracy, stability, and computational efficiency underscores its suitability for practical BCI systems, particularly where low-latency inference and rapid fine-tuning are essential.

Overall, the findings support transfer learning as an effective strategy for improving cross-participant robustness in MI-EEG classification under controlled subject-wise evaluation. These improvements should be interpreted as enhanced generalization within existing datasets rather than definitive proof of universal subject-independence.

Future work will therefore pursue three directions. First, we plan to validate the framework on larger and more diverse datasets to assess cross-laboratory and cross-session generalization. Second, integration with domain-adversarial and subject-invariant learning methods will be explored to further stabilize representations across heterogeneous subjects. Third, online and adaptive fine-tuning strategies will be investigated to evaluate real-time deployment feasibility and user-specific calibration efficiency.

By systematically analyzing fine-tuning depth, activation selection, and transfer strategies, this work provides practical design guidance for robust MI-EEG decoding and contributes to the incremental progress toward reliable and accessible brain-computer interfaces.

## Data Availability

Publicly available datasets were analyzed in this study. This data can be found at: https://bbci.de/competition/iv/download/.
